# RBM15B enhancing ITGA1 mRNA stability can accelerate glioblastoma tumorigenesis via the PI3K–Akt pathway

**DOI:** 10.1007/s12672-026-05064-3

**Published:** 2026-04-22

**Authors:** Wei Zhu, Yiheng Jin, Shaobing Wan

**Affiliations:** Emergency Department, Wuhan Third Hospital, No. 241, Pengliuyang Road, Wuchang District, Wuhan, 430060 Hubei China

**Keywords:** Glioblastoma, RBM15B, ITGA1, PI3K-Akt, N6-methyladenosine

## Abstract

**Background:**

N6-methyladenosine (m^6^A) modification is a key regulatory mechanism involved in the tumorigenesis of glioblastoma (GBM). The oncogenic role of m^6^A writer RNA-binding motif protein 15B (RBM15B) has been confirmed in multiple cancers. However, its role and regulatory mechanism in GBM remain unclear. This study aimed to explore the role of RBM15B interacting with integrin alpha-1 (ITGA1) in GBM.

**Methods:**

GEPIA, GEO DataSets and CGGA database were investigated to confirm the levels of RBM15B and the correlation between RBM15B and ITGA1 in GBM. Quantitative reverse transcription polymerase chain reaction (qRT-PCR) was performed to further verify RBM15B and ITGA1 levels in GBM samples. The effects of RBM15B and ITGA1 on GBM cells were determined through cell functional experiments and an animal assay. The interaction between RBM15B and ITGA1 was detected using qRT-PCR, methylated RNA immunoprecipitation assay, and actinomycin D therapy. The effects of RBM15B and ITGA1 on phosphoinositide 3-kinase–protein kinase B (PI3K–Akt) pathway-related proteins were assessed by Western blotting.

**Results:**

RBM15B was highly expressed in GBM, and RBM15B downregulation suppressed GBM cell proliferation, migration, invasion, and tumor growth. ITGA1 mRNA stability decreased by reducing its m^6^A modification level by downregulating RBM15B. Furthermore, ITGA1 overexpression induced GBM cell malignancy by activating the PI3K–Akt pathway; however, downregulating RBM15B partly reversed the effects of ITGA1 overexpression.

**Conclusion:**

This study uncovers a novel mechanism by which RBM15B promotes ITGA1 mRNA stability through m^6^A modification, leading to the activation of the PI3K–Akt pathway and promoting GBM progression.

**Supplementary Information:**

The online version contains supplementary material available at 10.1007/s12672-026-05064-3.

## Introduction

Glioblastoma (GBM) is the most aggressive and lethal primary brain tumor [1]. Despite multimodal treatment approaches, such as surgical resection, radiotherapy, and chemotherapy, the median survival of patients with GBM is approximately 10–16 months owing to its high invasiveness and distant metastasis [2–6]. This dismal prognosis underscores the urgent need to elucidate the molecular mechanisms driving the tumorigenesis of GBM and identify crucial therapeutic targets.

Recent studies have highlighted the regulatory role of N6-methyladenosine (m^6^A), the most abundant RNA modification in eukaryotes, in cancer progression [7–9]. m^6^A modification is dynamically deposited by a “writer” (e.g., METTL3 and WTAP), removed by an “eraser” (e.g., FTO and ALKBH5), and recognized by a “reader” (e.g., YTHDF1/2), which collectively regulate mRNA stability, splicing, transport, and translation [10–12]. Among m^6^A writers, RNA-binding motif protein 15B (RBM15B) was identified as a key player in tumorigenesis. For example, RBM15B facilitated prostate cancer proliferation by inducing PCNA m^6^A modification [13]. RBM15B was also reported to trigger the tumorigenesis of triple-negative breast cancer by influencing the m^6^A levels of serine and glycine metabolic genes [14]. RBM15B overexpression in hepatocellular carcinoma could enhance hepatocellular carcinoma cell malignancy by regulating TRAM2 mRNA stability in an m^6^A-modified manner [15]. Although RBM15B has been studied in multiple cancers, its function in GBM remains poorly understood.

Integrins are transmembrane receptors that mediate cell–extracellular matrix (ECM) interactions, and the active integrin complex contains alpha and beta subunits [16]. Integrin alpha-1 (ITGA1), a member of the integrin family, has emerged as a critical mediator of tumor cell adhesion and migration by interacting with the ECM to regulate intercellular signaling transduction [17]. Recently, the oncogenic role of ITGA1 has been widely established in pancreatic cancer [18], breast cancer [19], and hepatocellular carcinoma [20]. In GBM, circ_0043949-regulated ITGA1 was verified as an oncogene [21]. However, the mechanism regulating ITGA1 expression in GBM, particularly m^6^A-dependent mRNA modification, remains unexplored.

The phosphoinositide 3-kinase/protein kinase B (PI3K–Akt) pathway, an oncogenic signaling pathway, is frequently hyperactivated in GBM through alterations in genes such as *NRBP1* [22], *SLC25A32* [23], and *NQO1* [24]. Emerging evidence reveals that ITGA1 can activate the PI3K–Akt signaling pathway to participate in tumorigenesis. For example, ITGA1 was reported to promote sunitinib resistance by inducing the activation of the PI3K–Akt pathway in renal cell carcinoma [25]. In glioma, ITGA1 as a downstream of hsa_circ_0110757 activated the PI3K–Akt signaling pathway to regulate temozolomide resistance [26]. However, whether ITGA1 engages the PI3K–Akt pathway in GBM and whether its expression is regulated by RBM15B-mediated m^6^A methylation remains unexplored.

This study aimed to explore the mechanism of RBM15B as a critical m^6^A writer to drive the tumorigenesis of GBM by regulating ITGA1 mRNA stability and the PI3K–Akt pathway. The results may establish RBM15B as a critical regulator of ITGA1 and highlight the RBM15B–ITGA1–PI3K–Akt axis as a promising therapeutic target in GBM.

## Materials and methods

### Databases analysis

The GEPIA-GBM database, including 163 tumor and 207 normal samples, was employed to analyze RBM15B expression in GBM and the RBM15B-correlated genes in GBM. GSE100675 downloaded from another database, GEO DataSets, was an mRNA microarray including three GBM and three paired nontumor samples for screening upregulated genes in GBM, with *P* < 0.01 and logFC > 2 as screening criteria. Finally, the Gene Ontology (GO) and Kyoto Encyclopedia of Genes and Genomes (KEGG) enrichment of the upregulated genes were examined by STRING 12.0. Chinese Glioma Genome Atlas (CGGA) database was used to analyze the correlation between RBM15B and ITGA1 in GBM samples.

### Clinical samples

GBM and paired normal tissues were collected from 20 patients diagnosed with GBM in our hospital by pathologists based on the diagnostic standards of the World Health Organization. Patients or their families provided informed consent, and the ethics committee of Wuhan Third Hospital authorized this study (approval number: KY2024-013-01). Table 1 shows the specific characteristics of the patients.

**Table 1:** Characteristics of GBM patients (*n* = 20)

Characteristics	No. (%)
Age (years)	
≤55	11 (50.0)
>55	9 (16.7)
Sex	
Male	13 (65.0)
Female	7 (35.0)
Main symptoms	
Epilepsy	3 (15.0)
Intracranial. hypertension	9 (45.0)
Cystic change	5 (25.0)
Necrosis	3 (15.0)
Comorbidities	
Yes	18 (90.0)
No	2 (10.0)

### Cell culture and PI3K–Akt inhibitor treatment

Human GBM cell lines LN229 (BNCC341218) and T98G (BNCC338721) were purchased from BeNa Culture Collection (China), whereas the human normal astrocyte cell line NHA (BFN60808805) was purchased from BlueFBio (China). All cell lines were cultured in Dulbecco’s Modified Eagle Medium (BlueFBio) and 10% fetal bovine serum (FBS, BlueFBio) at 5% CO_2_ and 37℃. PI3K–Akt inhibitor LY294002 (20 µM, Beyotime Biotechnology, China) was added to the GBM cells and incubated for 24 h.

### Quantitative real-time reverse transcription polymerase chain reaction (qRT-PCR)

Total RNA was isolated using TRIzol Up (ET111-01-V2, TransGen Biotech, China) according to the manufacturer’s instructions. Then, RNA was reverse-transcribed to cDNA using 1st Strand cDNA Synthesis SuperMix (11141ES, Yeasen, China), and qRT-PCR was performed using qPCR SYBR Master Mix (11185ES03, Yeasen). mRNA expression was calculated using 2^−ΔΔCT^ with GAPDH as the endogenous control. Table 2 lists the primer sequences for qRT-PCR.

**Table 2:** The primers used for qRT-PCR

Gene	Sequence (5′-3′)	
RBM15B	Forward	TGGTAACCTGGACCACAGCGTA
	Reverse	GGTTCTGGAACTTGAGGAAGGC
ITGA1	Forward	GGTGCTTATTGGTTCTCCGTTAG
	Reverse	CTCCTTTACTTCTGTGACATTGGG
METTL3	Forward	CTATCTCCTGGCACTCGCAAGA
	Reverse	GCTTGAACCGTGCAACCACATC
METTL14	Forward	CTGAAAGTGCCGACAGCATTGG
	Reverse	CTCTCCTTCATCCAGATACTTACG
WTAP	Forward	GCAACAACAGCAGGAGTCTGCA
	Reverse	CTGCTGGACTTGCTTGAGGTAC
GAPDH	Forward	AACGAACACAAGTTACCTATC
	Reverse	ACATGAGGGAAACCGAGGG

### Cell transfection

Two siRNAs targeting RBM15B (GenePharma, China) were transfected into two GBM cell lines (LN229 and T98G) using the Lipo2000 reagent (Invitrogen, USA) for silencing RBM15B. The ITGA1 overexpression vector (GenePharma) was transfected into LN229 and T98G using the Lipo2000 reagent for upregulating ITGA1.

### GBM cell proliferation detection

GBM cell proliferation was detected using the Cell Counting Kit-8 (CCK8, Yeasen) and the 594 Click-iT EdU Imaging Kit (Yeasen). In CCK8, 2000 GBM cells were seeded onto 96-well plates. After transfection for 0, 24, 48, and 72 h, the optical density of each well was measured at 450 nm after adding 10 µL of the CCK8 solution into each well for 1.5 h incubation. As for the 5-ethynyl-2’-deoxyuridine (EdU) assay, 2000 GBM cells seeded onto a 96-well plate were subjected to cell transfection for 24 h. Then, the cells were fixed with 4% paraformaldehyde, and stained by 1 × Click-iT EdU reaction mixture and Hoechst 33,342. The cell images were photographed using a fluorescence microscope (Olympus, Japan).

### GBM cell migration detection

A wound-healing assay was performed to detect GBM cell migration. After transfection, GBM cells were seeded onto a 6-well plate. When the cells grew to > 90% confluence, the cell monolayers were scraped using a 200-µL pipette tip. The wound images at 0 and 24 h were captured using a light microscope (Olympus).

### GBM cell invasion detection

The Transwell assay was conducted to detect GBM cell invasion. After coating the top chamber of the Transwell using Matrigel overnight, the transfected GBM cells with serum-free medium were added to the top chamber, and the medium mixed with 10% FBS was added to the bottom chamber. After 24 h of incubation, GBM-invasive cells were fixed, stained, and photographed to measure the number of invasive cells.

### Animal experiment

Lentivirus-packaged RBM15B shRNA (GenePharma, China) was transfected into T98G cells for constructing stably RBM15B low-expressed GBM cells. Then, the transfected T98G cells (1 × 10^7^) were subcutaneously inoculated into BALB/c nude mice (male, 5 weeks old; Cyagen, China), with five mice per group. A caliper was used to measure the tumor volume every 7 days using the formula: volume = (length × width²)/2. The tumors were removed, photographed, and weighed, and Ki67 staining was performed to evaluate the proliferative activity after the mice were euthanized at day 35 using 200 mg/kg pentobarbital sodium by tail vein injection. The animal experiment was approved by the ethics committee of Wuhan Third Hospital with tumor size < 2000 mm^3^ and tumor weight < 10% of normal nude mice weight (approval number: 2024022).

### Methylated RNA immunoprecipitation (MeRIP) assay

The MeRIP assay was performed to detect the m^6^A level of ITGA1 using the GenSeq m^6^A MeRIP Kit (GenSeq, China). Total RNA (18 µg) isolated from GBM cells was fragmented to an average size of 100–200 nucleotides using fragmentation buffer. Then, the fragmented RNA was incubated with the magnetic beads preincubated with m6A or IgG antibody at 4 °C for 2 h with gentle rotation. After extensive washing to remove non-specifically bound RNA, the immunoprecipitated RNA was eluted and purified. The enrichment of ITGA1 mRNA in the immunoprecipitated fraction was quantified by qRT-PCR. Primers for ITGA1 were designed to amplify regions containing the predicted m^6^A sites. The data were normalized to input RNA to account for variations in RNA abundance, and the IgG control was included to evaluate background binding. The results were expressed as fold enrichment relative to the IgG.

### RIP-qPCR assay

GBM cells (1 × 10^7^) were collected by centrifugation at 1,500 rpm for 5 min at 4 °C, and the RIP assay was performed by incubating with magnetic beads conjugated with 5 µg of anti-IgG antibody, anti-RBM15B antibody or anti-WTAP antibody using the Magna RIP Kit (Merck, USA). Finally, ITGA1 enrichment was measured by qRT-PCR.

### Luciferase assay

Using RMBase v2.0 to predict the m^6^A site on ITGA1 mRNA, the ITGA1 wild-type vector (ITGA1-WT) containing the m^6^A site and the ITGA1 mutant vector (ITGA1-MUT) not containing the m^6^A site were constructed by GenePharma and co-transfected with si-RBM15B into GBM cells. After 24 h of incubation, luciferase activity was detected using a Luciferase Reporter Assay Kit (Beyotime Biotechnology).

### ITGA1 mRNA stability detection

ITGA1 mRNA stability was detected using actinomycin D (Beyotime Biotechnology) to treat GBM cells. Two GBM cell lines (LN229 and T98G) after transfection were exposed to actinomycin D for 0, 3, and 6 h. At each indicated time point, the GBM cells were collected to isolate RNA for qRT-PCR to detect ITGA1 expression.

### Western blotting

RIPA Lysis Buffer (RM02998, ABclonal, China) was added to the cells for protein extraction. Then, after the concentrations were determined, proteins were separated by sodium dodecyl sulfate polyacrylamide gel electrophoresis, blotted onto polyvinylidene fluoride membranes, blocked by 5% skim milk, and incubated with primary antibodies, including PI3K antibody (A4992, ABclonal), phospho-PI3K antibody (p-PI3K, AP0854, ABclonal), Akt antibody (A17909, ABclonal), phospho-Akt (p-Akt, AP1208, ABclonal), phospho-mTOR antibody (p-mTOR, AP1413, ABclonal), mTOR antibody (A2445, ABclonal), and GAPDH antibody (AC027, ABclonal). After overnight incubation, the protein in the membranes was interacted with the secondary antibody (AS014, ABclonal) for 3 h and visualized using the ECL Enhanced Plus Kit (RM00021P, ABclonal).

### Data analysis

All data from three experiments were analyzed by GraphPad Prism 10.1.2 and shown as mean ± SD. Differences were analyzed using the t-test (two groups) and one-way analysis of variance (multiple groups). *P* < 0.05 indicated a significant difference.

## Results

### RBM15B with high GBM expression

**Fig. 1:**
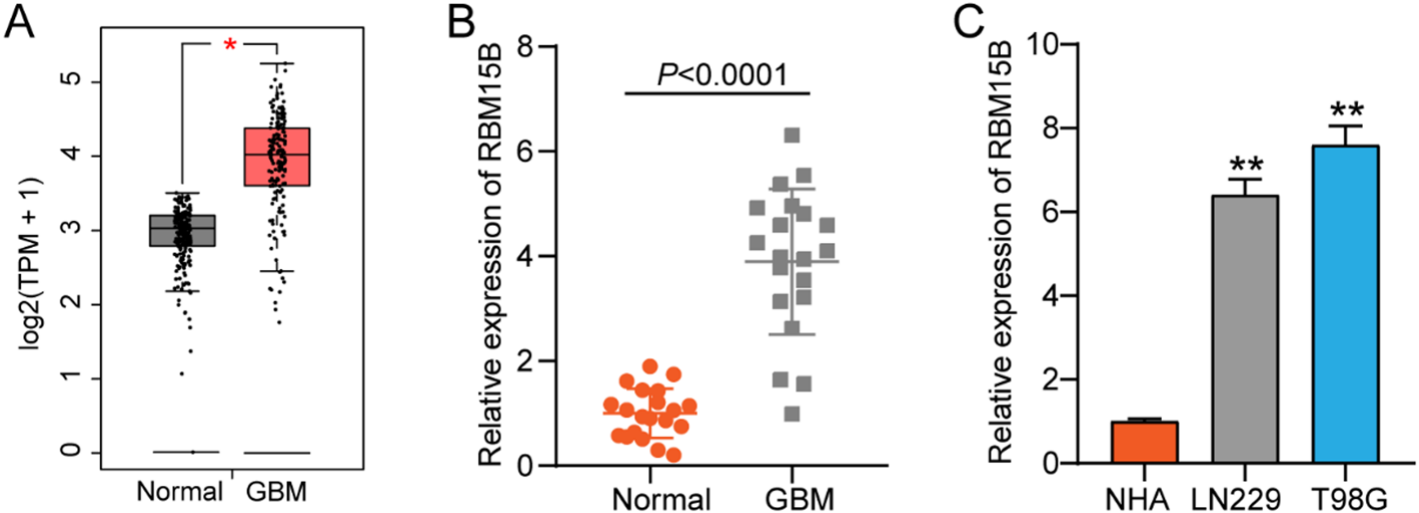
RBM15B with high expression in GBM. **A** GEPIA-GBM database showing the RBM15B expression in tumor and normal samples. Data are based on publicly available RNA-seq datasets (Normal, *n* = 207; GBM, *n* = 163). *P < 0.01 was determined using the method implemented by GEPIA (ANOVA). These data are summarized from public datasets and are distinct from experimental measurements. **B** qRT-PCR detected the RBM15B expression in GBM and adjacent normal samples; *N* = 20. *P* < 0.0001 using the t-test. **C** qRT-PCR detected the RBM15B expression in GBM cell lines (LN229 and T98G) and the normal astrocyte cell line NHA; *n* = 3. **P < 0.001 using the one-way ANOVA. All experimental data are shown as mean ± SD. Each experiment was repeated three times

In the GEPIA-GBM database, RBM15B was overexpressed in tumor samples (Fig. 1A). After performing qRT-PCR in the clinical samples from our hospital, RBM15B expression also increased in the GBM samples (Fig. 1B). Similarly, RBM15B expression in GBM cell lines (LN229 and T98G) was upregulated compared with that in the normal astrocyte cell line NHA (Fig. 1C). Data from the GEPIA-GBM database and qRT-PCR proved that RBM15B was upregulated in GBM.

### RBM15B downregulation suppresses GBM cell malignancy

**Fig. 2:**
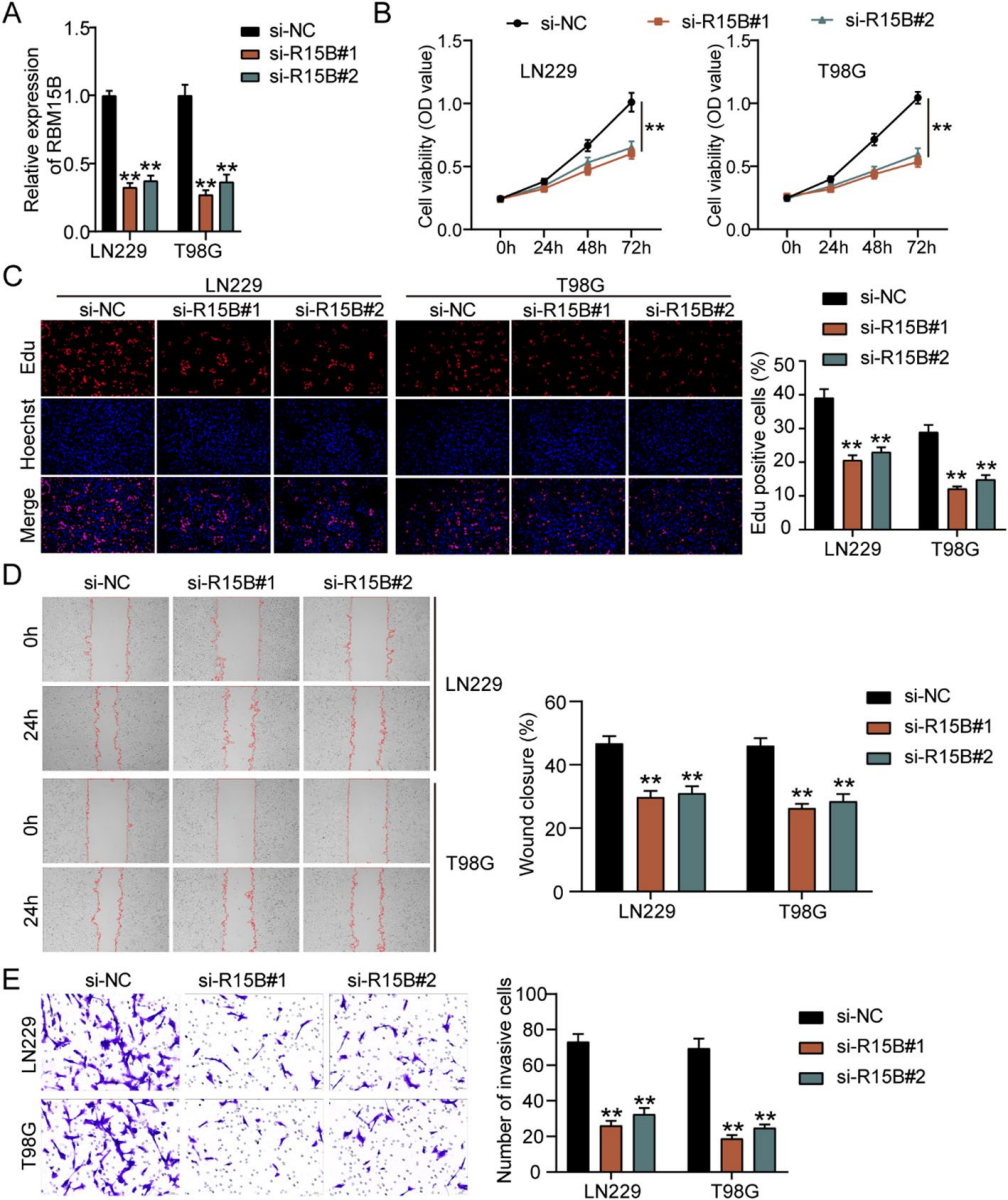
RBM15B downregulation suppresses GBM cell malignancy. **A** qRT-PCR detected the transfection efficiency of two siRNAs targeting RBM15B (si-R15B#1 and si-R15B#2) in GBM cells. **B** and **C** CCK8 and EdU assays detected GBM cell proliferation after the transfection of si-R15B#1 and si-R15B#2. **D** In wound-healing assays, GBM cell migration was assessed after the transfection of si-R15B#1 and si-R15B#2. **E** Transwell assay verified GBM cell invasion after the transfection of si-R15B#1 and si-R15B#2. **P < 0.001 vs. si-NC using the one-way ANOVA; *n* = 3. All data are shown as mean ± SD. Each experiment was repeated three times

To verify the function of RBM15B in GBM, two siRNAs targeting RBM15B (si-R15B#1 and si-R15B#2), which were used for downregulating RBM15B, were transfected into GBM cells. qRT-PCR showed that RBM15B expression was reduced by approximately 70% in GBM cells after transfection with si-R15B#1 and si-R15B#2 (Fig. 2A). Then, the cell proliferation ability detected by the CCK8 and EdU assays indicated that downregulating RBM15B in GBM cells could prevent GBM cells from proliferating (Fig. 2B and C). Regarding cell migration, the wound closure rate decreased in GBM cells after transfection of two siRNAs targeting RBM15B, suggesting that downregulating RBM15B inhibited GBM cell migration (Fig. 2D). Moreover, the Transwell assay revealed that downregulating RBM15B also attenuated GBM cell invasion (Fig. 2E). These results confirmed the inhibitory function of RBM15B downregulation in GBM cells.

### RBM15B downregulation suppresses tumor growth in vivo

**Fig. 3:**
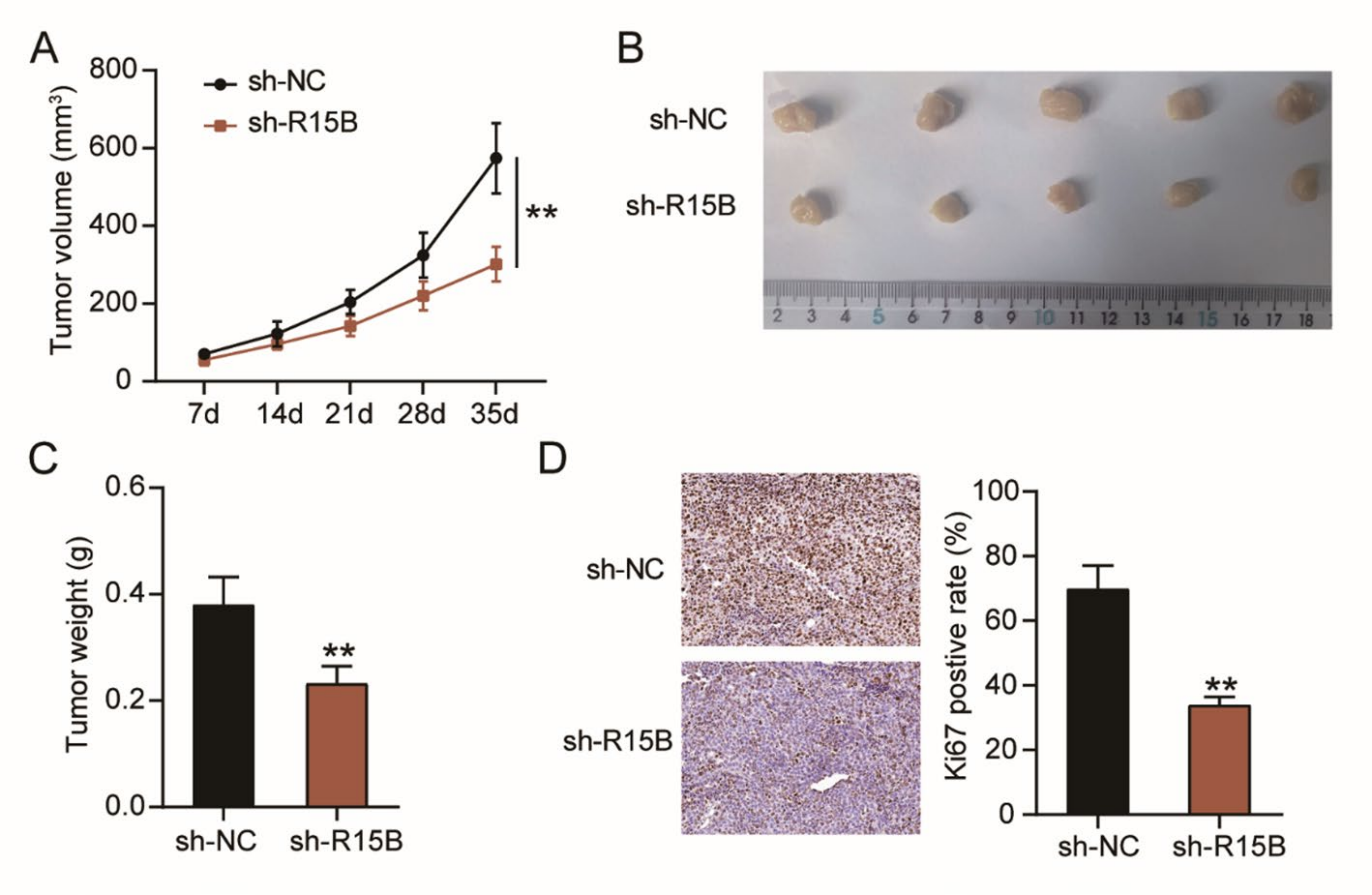
RBM15B downregulation suppresses tumor growth in vivo. Lentivirus packaging RBM15B knockdown vector (sh-R15B) was used to transfect T98G cells and then construct stably low expression of RBM15B GBM cells, which were subcutaneously injected into BALB/c nude mice (*n* = 5/group). **A** A Vernier caliper was used to measure the tumor volume weekly. **B** Tumors from nude mice were photographed. **C** Tumor from nude mice were weighed after 35 days. **D** The Ki67-positive rate of tumors from nude mice was calculated by Ki67 staining. **P < 0.001 vs. sh-NC using t-test; *n* = 5. All data are shown as mean ± SD. Each experiment was repeated three times

Because the inhibitory effects of downregulating RBM15B were stronger in T98G cells (Fig. 2), a lentivirus-packaged RBM15B knockdown vector (sh-R15B) was transfected into T98G cells and subcutaneously injected into nude mice to confirm the effect of downregulating RBM15B on tumor growth in vivo. The results revealed that downregulating RBM15B reduced the tumor volume (Fig. 3A), size (Fig. 3B), and weight (Fig. 3C) and Ki67-positive rate (Fig. 3D). All the data proved that RBM15B downregulation could suppress tumor growth in vivo.

### RBM15B downregulation reduces ITGA1 mRNA stability by reducing the m6A level of ITGA1 in GBM cells

**Fig. 4:**
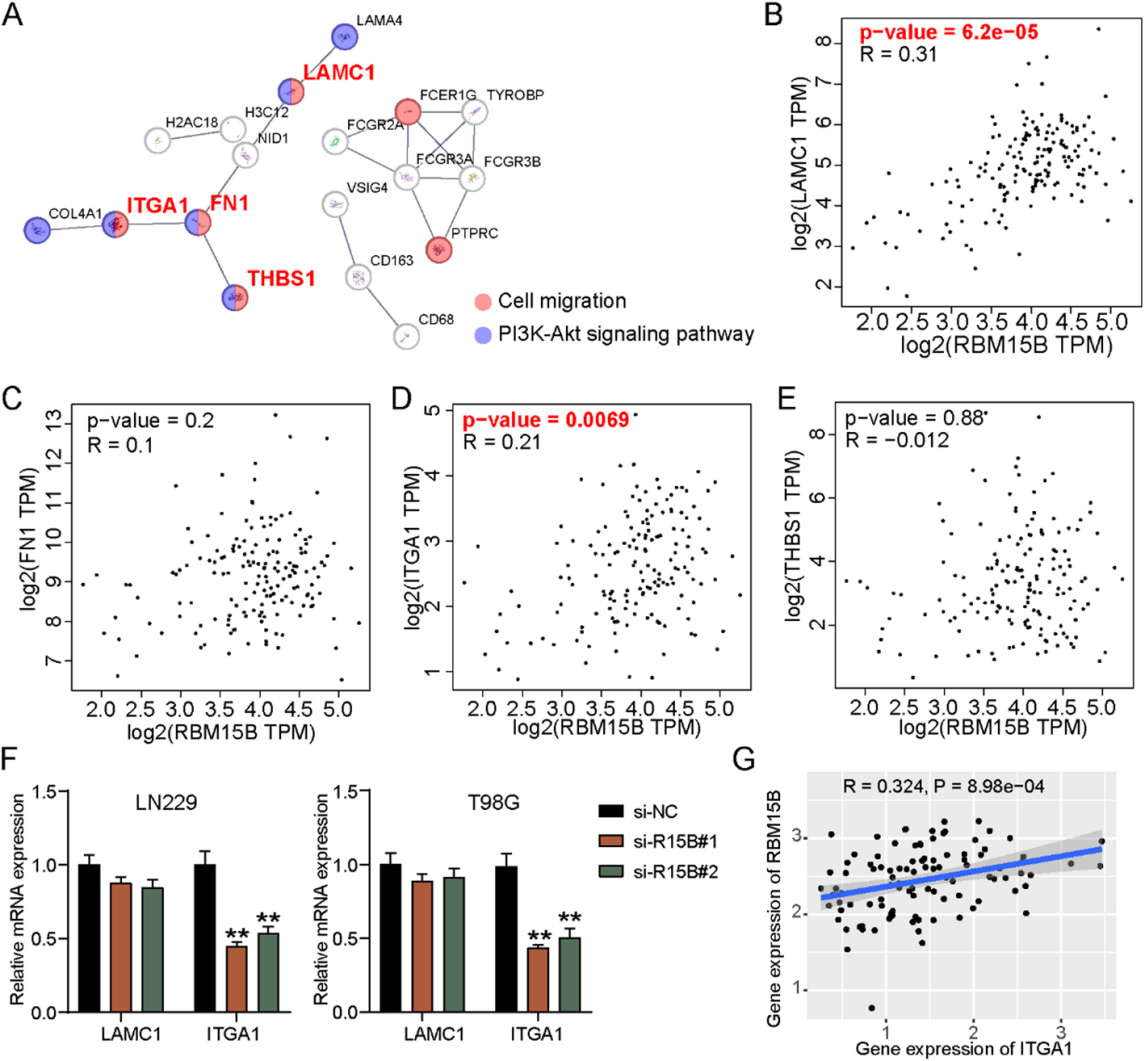
LAMC1 and ITGA1 may be positively regulated by RBM15B in GBM cells. **A** STRING was employed to analyze the GO and KEGG enrichment of 43 upregulated genes in GBM samples from an mRNA microarray GSE100675. **B–E** GEPIA-GBM database showed the correlation between RBM15B expression and LAMC1/FN1/ITGA1/THBS1 expression in GBM samples. **F** qRT-PCR detected the expression of LAMC1 and ITGA1 in GBM cells after the transfection of two siRNAs targeting RBM15B (si-R15B#1 and si-R15B#2); *n* = 3. **P<0.001 vs. si-NC using the one-way ANOVA. **G** CGGA database showed the correlation between RBM15B and ITGA1 expression in GBM samples

**Fig. 5:**
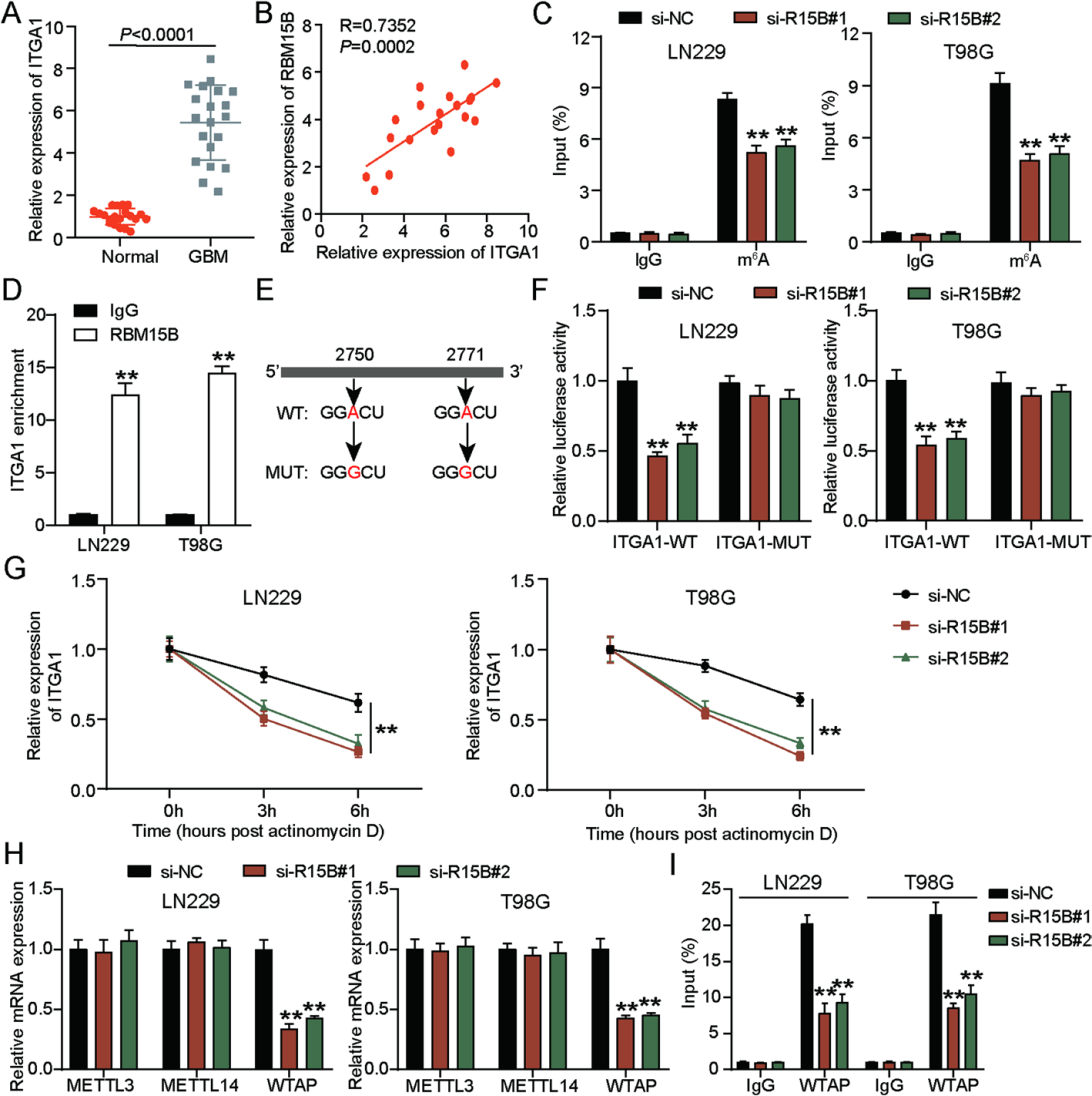
RBM15B downregulation reduced ITGA1 mRNA stability by reducing the m^6^A level of ITGA1 in GBM cells. **A** qRT-PCR detected the ITGA1 expression in GBM and adjacent normal samples; *n* = 20. *P* < 0.0001 using the t-test. **B** The correlation between ITGA1 expression and RBM15B expression in GBM samples was analyzed by Pearson correlation analysis. **C** The m^6^A level of ITGA1 in GBM cells was measured in MeRIP after the transfection of si-R15B#1 and si-R15B#2; *n* = 3. **P < 0.001 vs. si-NC using the one-way ANOVA. **D** In RIP-qPCR, the binding of RBM15B to ITGA1 mRNA was analyzed; *n* = 3. **P < 0.001 vs. IgG using the one-way ANOVA. **E** RMBase v2.0 showed the m^6^A site on ITGA1 mRNA. **F** In the luciferase assay, the specific m^6^A site within ITGA1 mRNA affected by RBM15B was identified; *n* = 3. **P < 0.001 vs. si-NC using the one-way ANOVA. **G** Actinomycin D treatment assay confirmed the change in ITGA1 mRNA stability in GBM cells after the transfection of si-R15B#1 and si-R15B#2; *n* = 3. **P < 0.001 vs. si-NC using the one-way ANOVA. **H** qRT-PCR detected the expression of METTL3, METTL14 and WTAP in GBM cells after the transfection of si-R15B#1 and si-R15B#2; *n* = 3. **P < 0.001 vs. si-NC using the one-way ANOVA. **I** RIP-qPCR assay confirmed the binding of WTAP to ITGA1 mRNA in GBM cells after the transfection of si-R15B#1 and si-R15B#2; *n* = 3. **P < 0.001 vs. si-NC using the one-way ANOVA. All data are shown as mean ± SD. Each experiment was repeated three times

After setting *P* < 0.01 and logFC > 2 as screening criteria, 43 upregulated genes in GBM samples were screened from GSE100675 and uploaded to STRING for GO and KEGG enrichment. As shown in Fig. 4A, four genes (*LAMC1*, *FN1*, *THBS1*, and *ITGA1*) showed correlation with cell migration and the PI3K–Akt signaling pathway. According to the data from the GEPIA-GBM database, only the expression levels of LAMC1 and ITGA1 were predicted to be correlated to RBM15B expression (Fig. 4B and E). The qRT-PCR data further confirmed that only ITGA1 expression was reduced in GBM cells after the transfection of RBM15B-knockdown plasmids (Fig. 4F). The data from CGGA database showed a significant positive correlation between RBM15B and ITGA1 expression in GBM samples (Fig. 4G). In the collected clinical samples, ITGA1 expression was found to be overexpressed in the GBM samples, and its expression was positively correlated with RBM15B expression in the GBM samples (Fig. 5A and B). The MeRIP assay further confirmed that downregulating RBM15B in GBM cells reduced the m^6^A level of ITGA1 (Fig. 5C). The RIP-qPCR assay demonstrated the binding of RBM15B to ITGA1 mRNA (Fig. 5D). The m^6^A site on ITGA1 mRNA was predicted by RMBase v2.0 (Fig. 5E). Using the luciferase assay, RBM15B silencing induced a decrease in the luciferase activity in the ITGA1-WT group, whereas RBM15B silencing did not affect the luciferase activity in the ITGA1-MUT group (Fig. 5F). In addition, ITGA1 mRNA stability was impaired in GBM cells after the transfection of RBM15B-knockdown plasmids (Fig. 5G). To further investigate the mechanism of RBM15B-mediated ITGA1 m^6^A modification, we examined key components of the m^6^A methyltransferase complex. qRT-PCR analysis revealed that RBM15B knockdown decreased WTAP expression, while no significant changes were observed in METTL3 or METTL14 expression (Fig. 5H). RIP-qPCR assay demonstrated that RBM15B silencing markedly reduced the recruitment of WTAP on ITGA1 mRNA (Fig. 5I). These findings indicated that RBM15B regulated ITGA1 m^6^A modification by facilitating the recruitment of WTAP to ITGA1 mRNA, thereby modulating its stability.

### RBM15B downregulation relieved the oncogenic role of ITGA1 overexpression in GBM cells

**Fig. 6:**
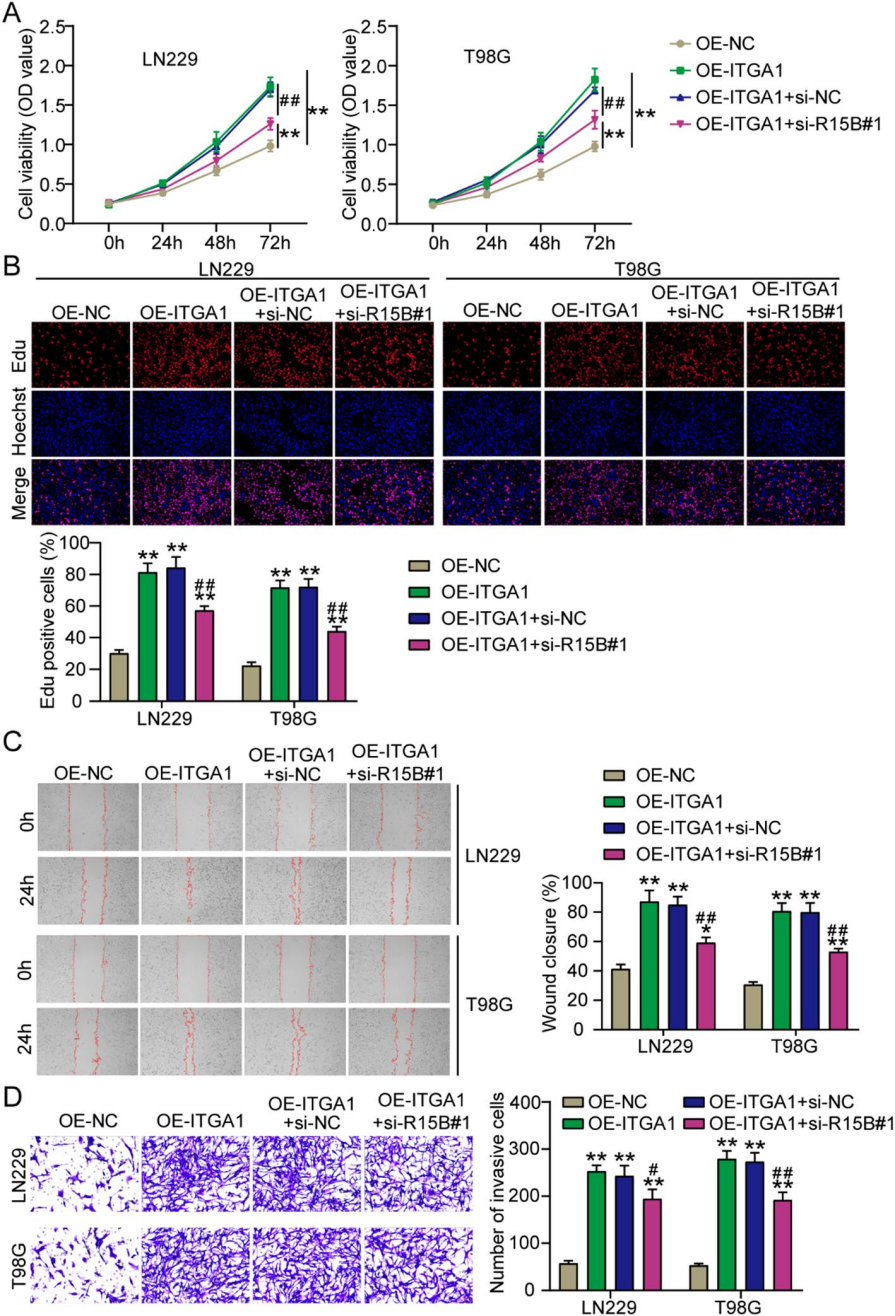
RBM15B downregulation relieved the promotive effect of ITGA1 overexpression on GBM cell malignancy. **A** and **B** In CCK8 and EdU assays, GBM cell proliferation was determined after the transfection of the ITGA1 overexpression vector (OE-ITGA1) and siRNA targeting RBM15B (si-R15B#1). **C** In the wound-healing assay, GBM cell migration was measured after the transfection of OE-ITGA1 and si-R15B#1. **D** Transwell assay verified GBM cell invasion after the transfection of OE-ITGA1 and si-R15B#1. *P < 0.05, **P < 0.001 vs. OE-NC and #*P* < 0.05, ##*P* < 0.001 vs. OE-ITGA1 + si-NC using the one-way ANOVA; *n* = 3. All data are shown as mean ± SD. Each experiment was repeated three times

After transfecting si-R15B#1 and the ITGA1 overexpression vector (OE-ITGA1) into GBM cells, ITGA1 overexpression enhanced GBM cell proliferation; however, this enhanced effect was partly impaired when GBM cells were co-transfected with si-R15B#1 (Fig. 6A and B). OE-ITGA1 also promoted GBM cell migration; however, si-R15B#1 partly inhibited the enhanced cell migration caused by OE-ITGA1 (Fig. 6C). As for cell invasion, ITGA1 overexpression led to an increase in GBM-invasive cells; however, RBM15B downregulation partly relieved this increase (Fig. 6D). Taken together, ITGA1 overexpression enhanced GBM cell malignancy, whereas RBM15B downregulation relieved ITGA1-induced GBM cell malignancy.

### RBM15B downregulation inhibited the activation of the PI3K–Akt pathway induced by ITGA1 overexpression in GBM cells

**Fig. 7:**
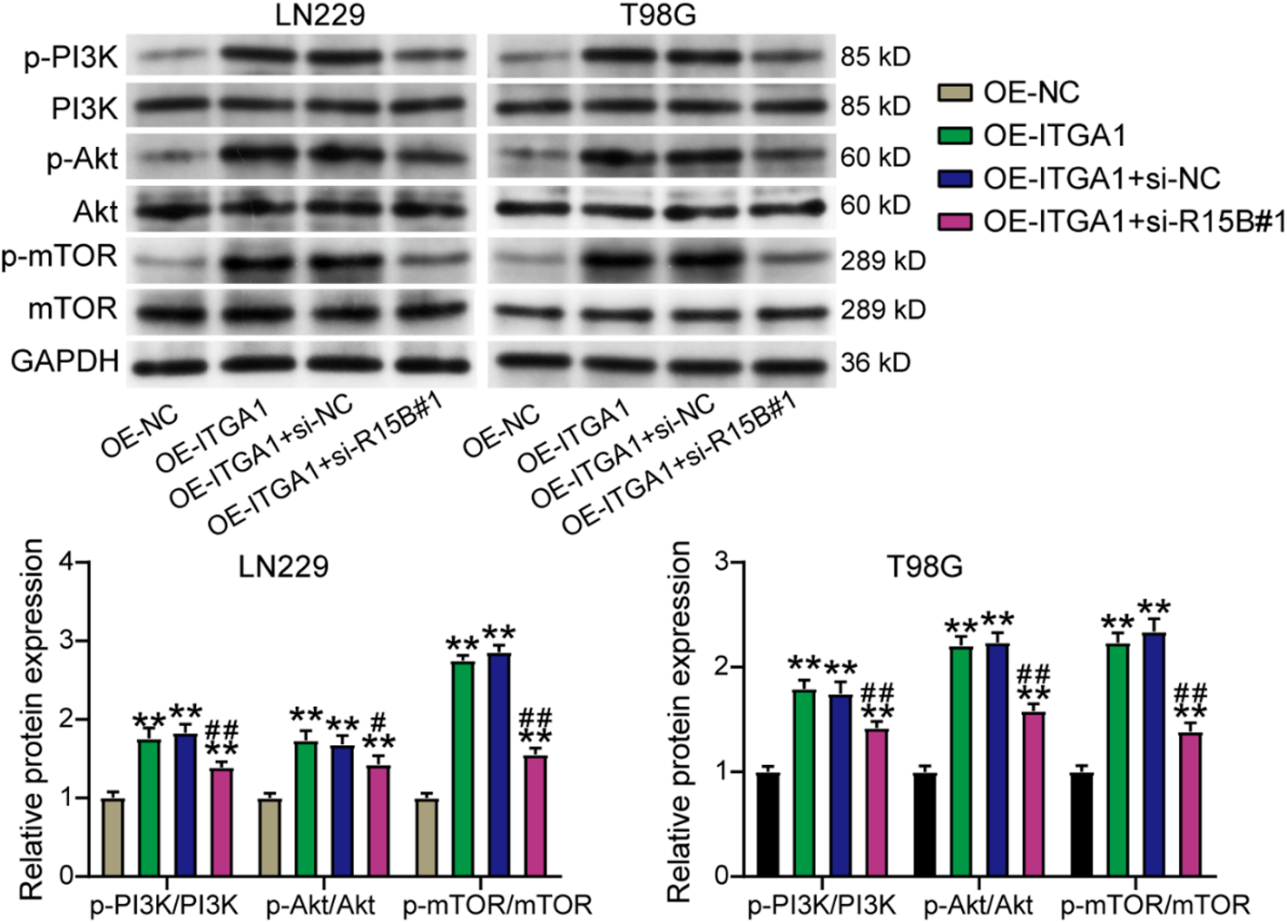
RBM15B downregulation inhibited the activation of the PI3K–Akt pathway induced by ITGA1 overexpression in GBM cells. In Western blotting, the key proteins of the PI3K–Akt pathway were detected in GBM cells after the transfection of the ITGA1 overexpression vector (OE-ITGA1) and siRNA targeting RBM15B (si-R15B#1). **P < 0.001 vs. OE-NC and #*P* < 0.05, ##*P* < 0.001 vs. OE-ITGA1 + si-NC using the one-way ANOVA; *n* = 3. All data are shown as mean ±SD. Each experiment was repeated three times

To further confirm the downstream pathway that could be regulated by the RBM15B–ITGA1 axis in GBM cells, Western blotting was performed to detect the key proteins of the PI3K–Akt pathway. The results showed that ITGA1 overexpression led to an increase in the protein levels of p-PI3K, p-Akt, and p-mTOR, whereas downregulating RBM15B reduced this increase caused by ITGA1 overexpression (Fig. 7). These data indicated that ITGA1 overexpression activated the PI3K–Akt pathway in GBM cells, whereas RBM15B downregulation impaired this activation.

### ITGA1 overexpression enhanced GBM cell malignancy via the PI3K–AKT pathway

**Fig. 8:**
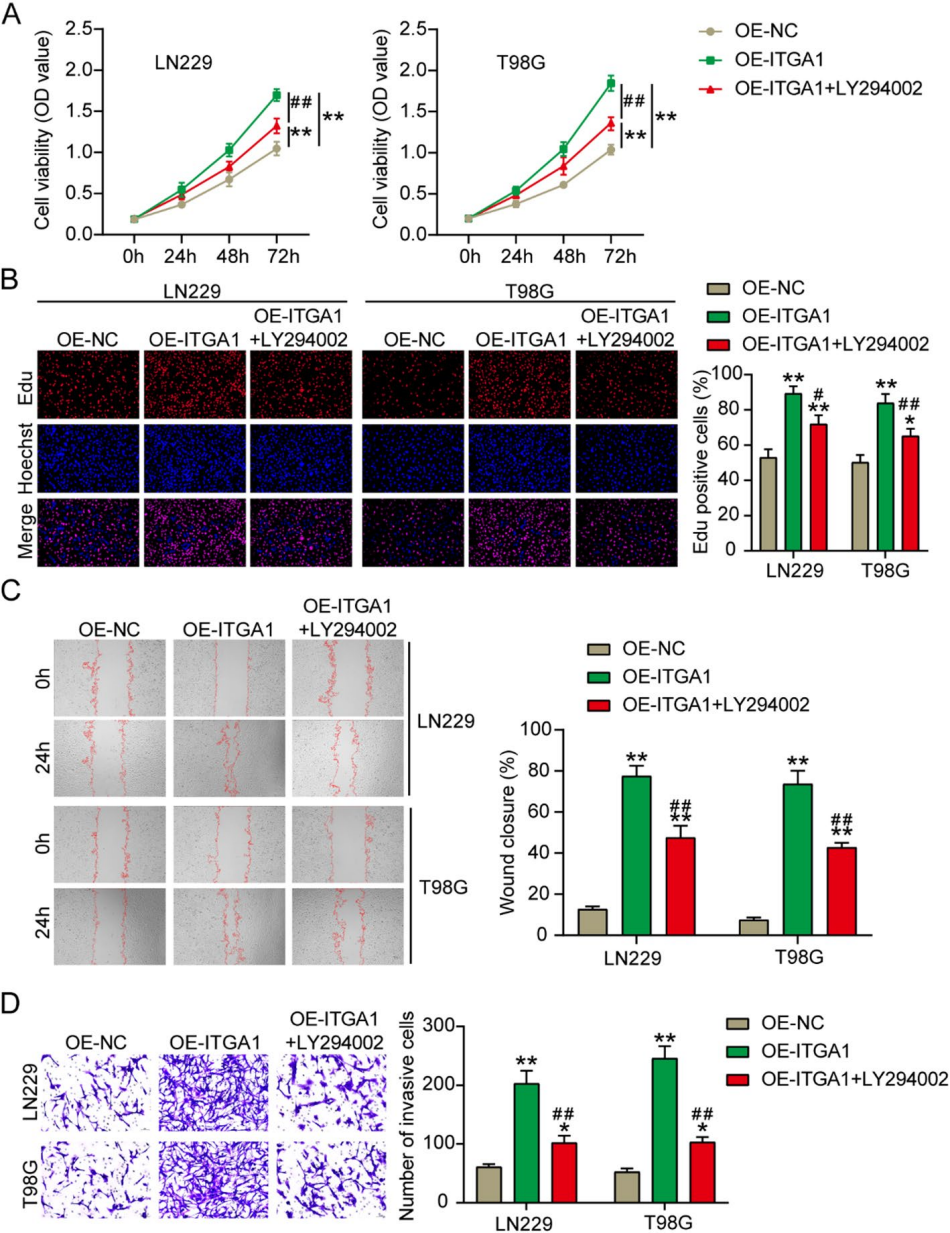
ITGA1 overexpression enhanced GBM cell malignancy via the PI3K–AKT pathway. **A** and **B** In CCK8 and EdU assays, GBM cell proliferation was determined after the transfection with the ITGA1 overexpression vector (OE-ITGA1) and PI3K–AKT inhibitor (LY294002) treatment. **C** In the wound-healing assay, GBM cell migration was measured after the transfection with OE-ITGA1 and LY294002 treatment. **D** Transwell assay verified GBM cell invasion after the transfection with OE-ITGA1 and LY294002 treatment. *P < 0.05, **P < 0.001 vs. OE-NC and #*P* < 0.05, ##*P* < 0.001 vs. OE-ITGA1 using the one-way ANOVA; *n* = 3. All data are shown as mean ± SD. Each experiment was repeated three times

To further prove the mechanism of ITGA1 overexpression on GBM cell malignancy via the PI3K–AKT pathway, functional rescue experiments were performed using the PI3K–AKT inhibitor LY294002 to treat GBM cells. CCK8 and EdU assays revealed that LY294002 reduced the enhanced GBM cell proliferation induced by ITGA1 overexpression (Fig. 8A and B). Regarding GBM cell migration and invasion, LY294002 relieved the promotive effects of ITGA1 overexpression (Fig. 8C and D). These results indicated that ITGA1 overexpression enhanced GBM cell malignancy via the PI3K–AKT pathway.

## Discussion

GBM is the most aggressive primary brain tumor in adults and is characterized by rapid proliferation, extensive infiltration, and resistance to conventional therapies [27, 28]. Understanding the molecular mechanisms underlying GBM progression is essential to identify novel therapeutic targets. This study elucidates a novel molecular mechanism by which RBM15B enhances ITGA1 mRNA stability through m^6^A modification, which hastens GBM tumorigenesis via the PI3K–Akt signaling pathway. These results collectively indicate RBM15B as a potential therapeutic target for GBM and provide insights into the intricate regulatory network of the RBM15B–ITGA1–PI3K–Akt axis in GBM.

m^6^A, a modification in eukaryotic mRNA, plays a pivotal role in GBM [29–31]. For example, METTL3, an m^6^A writer, was upregulated in GBM to promote tumorigenesis by inducing ADAR1 m^6^A modification [32]. RBM15B is also an m^6^A writer; however, its role in GBM has not been reported. In previous studies, RBM15B was found to facilitate tumorigenesis in prostate cancer by mediating PCNA m^6^A modification [13], triple-negative breast cancer by mediating the m^6^A modification of serine and glycine metabolic genes [14], and hepatocellular carcinoma by mediating TRAM2 m^6^A modification [15]. Therefore, RBM15B might play an oncogenic role in GBM, which was worthy of investigation. Through in vitro and in vivo experiments, this study confirmed the high expression of RBM15B in GBM, and silencing RBM15B suppressed GBM cell proliferation, migration, invasion, and tumor growth. These results proved that RBM15B is a critical tumor promoter in GBM. As RBM15B has been implicated in m^6^A-mediated post-transcriptional modification in other cancers, this study further identified ITGA1 as a direct downstream target of RBM15B. Notably, GEPIA-GBM database analysis revealed a relatively weak correlation between RBM15B and ITGA1 expression (*R* = 0.21, *P* = 0.0069). However, validation in an independent cohort from the CGGA database demonstrated a consistent positive correlation (*R* = 0.324, *P* = 8.98 × 10^− 4^), and our qRT-PCR analysis of clinical GBM samples revealed a markedly stronger association (*R* = 0.7352, *P* = 0.0002). Although the strength of correlation varies across datasets, the direction of association remains consistent, supporting a reproducible link between RBM15B and ITGA1 expression in GBM. The observed variability in correlation coefficients may reflect differences in cohort composition, sample size, data acquisition platforms, and normalization strategies and biological heterogeneity. In particular, bulk RNA-sequencing datasets such as GEPIA and CGGA may be influenced by tumor purity, stromal content, and intratumoral cellular diversity, which can attenuate gene–gene correlations. In contrast, our cohort, analyzed under controlled experimental conditions, may better capture the intrinsic relationship between RBM15B and ITGA1 expression. This discrepancy highlights the importance of validating bioinformatics predictions with experimental data and emphasizes the importance of patient-specific molecular profiling in GBM research. In addition, RBM15B has been reported as an m^6^A “writer” that recruits the m^6^A methyltransferase complex (consisting of METTL3, METTL14, and WTAP) to specific RNA substrates rather than directly catalyzing methylation [33, 34]. In our study, we demonstrate that RBM15B knockdown reduced WTAP expression without affecting METTL3 or METTL14 levels in GBM cells, and silencing RBM15B decreased the recruitment of WTAP on ITGA1 mRNA. Overall, our findings suggest that RBM15B promotes GBM progression by stabilizing ITGA1 mRNA via WTAP recruitment-dependent method, offering new insights into the epitranscriptomic regulation of GBM.

ITGA1, an integrin family member, mediates cell adhesion, migration, and signal transduction and has been associated with tumor progression. Recently, ITGA1 expression was found to be higher in low-grade gliomas than in normal samples, and its overexpression enhanced glioma cell invasion [35]. Regarding the regulatory mechanism of ITGA1, previous studies have focused on ITGA1 as the downstream of hsa_circ_0110757/miR-1298-5p or hsa_circ_0043949/miR-876-3p to promote glioma or GBM cell malignancy [21, 26]. The present results revealed that ITGA1 overexpression promotes GBM cell proliferation, migration, and invasion, which was consistent with the results of previous studies. However, the present study confirmed the RBM15B-mediated stabilization of ITGA1 mRNA via an m^6^A modification method, which put forward a novel post-transcriptional regulatory mechanism of ITGA1 in GBM.

In exploring the downstream signaling pathway, bioinformatic analysis was conducted to perform KEGG enrichment, which revealed the PI3K–Akt pathway was the downstream pathway of ITGA1. Literature review results indicated that the PI3K–Akt pathway is a well-established driver of tumor survival, growth, and chemoresistance in GBM [36–38]. NRBP1, SLC25A32, and NQO1 could activate the PI3K–Akt pathway to drive GBM progression [22–24], suggesting that this pathway could be regulated by multiple oncogenes in GBM. The present study demonstrates that RBM15B downregulation inhibits the activation of the PI3K–Akt pathway induced by ITGA1 overexpression in GBM. These results provide compelling evidence that RBM15B promotes GBM progression, at least in part, by ITGA1-mediated activation of the PI3K–Akt pathway.

This study identifies the RBM15B–ITGA1–PI3K–Akt axis as a novel oncogenic regulator in GBM; however, some limitations should be addressed in further investigations. First, although our data suggest that RBM15B enhances ITGA1 mRNA stability through m^6^A modification, the rescue experiments show that RBM15B knockdown partly reverses the effects of ITGA1 overexpression. It strongly suggests that RBM15B has other important mRNA targets. Future studies identifying other RBM15B-regulated mRNAs and their functional roles in GBM could provide a more comprehensive understanding of the oncogenic functions of RBM15B. Second, the current study focuses on a single downstream target selected based on functional relevance and bioinformatics screening. A more comprehensive characterization of RBM15B-mediated regulatory networks, such as through transcriptome-wide approaches including RNA sequencing combined with MeRIP-seq, would provide deeper insight into its global role in GBM progression. Third, although LN229 and T98G cell lines were used in our study due to their well-characterized molecular features, GBM is highly heterogeneous, and cell line-specific effects cannot be ruled out. Future studies validating RBM15B expression and function in additional GBM models, such as patient-derived xenografts or primary tumor cultures, would further strengthen the robustness and generalizability of the findings. Additionally, the present findings highlight RBM15B as a potential therapeutic target. Future studies should determine the therapeutic efficacy of PI3K–Akt pathway inhibitors or m^6^A modulators in RBM15B-driven GBM models.

Taken together, this study uncovers a novel mechanism by which RBM15B enhances ITGA1 mRNA stability through m^6^A modification, leading to the activation of the PI3K–Akt pathway and promoting GBM progression. This study not only elucidates the molecular mechanism of RBM15B-mediated ITGA1 stability in GBM but also highlights that targeting the RBM15B–ITGA1–PI3K–Akt axis may represent a promising avenue for the development of effective therapies for GBM.

## Supplementary Information

Below is the link to the electronic supplementary material.

Supplementary Material 1.

## Data Availability

The datasets generated during and/or analyzed during the current study are available from the corresponding author on reasonable request.
