# Supplementary figures and images for: RBM15B enhancing ITGA1 mRNA stability can accelerate glioblastoma tumorigenesis via the PI3K–Akt pathway
Source: Discov Oncol. 2026 Apr 22;17:854. doi: 10.1007/s12672-026-05064-3 (PMC13237321; doi:10.1007/s12672-026-05064-3)

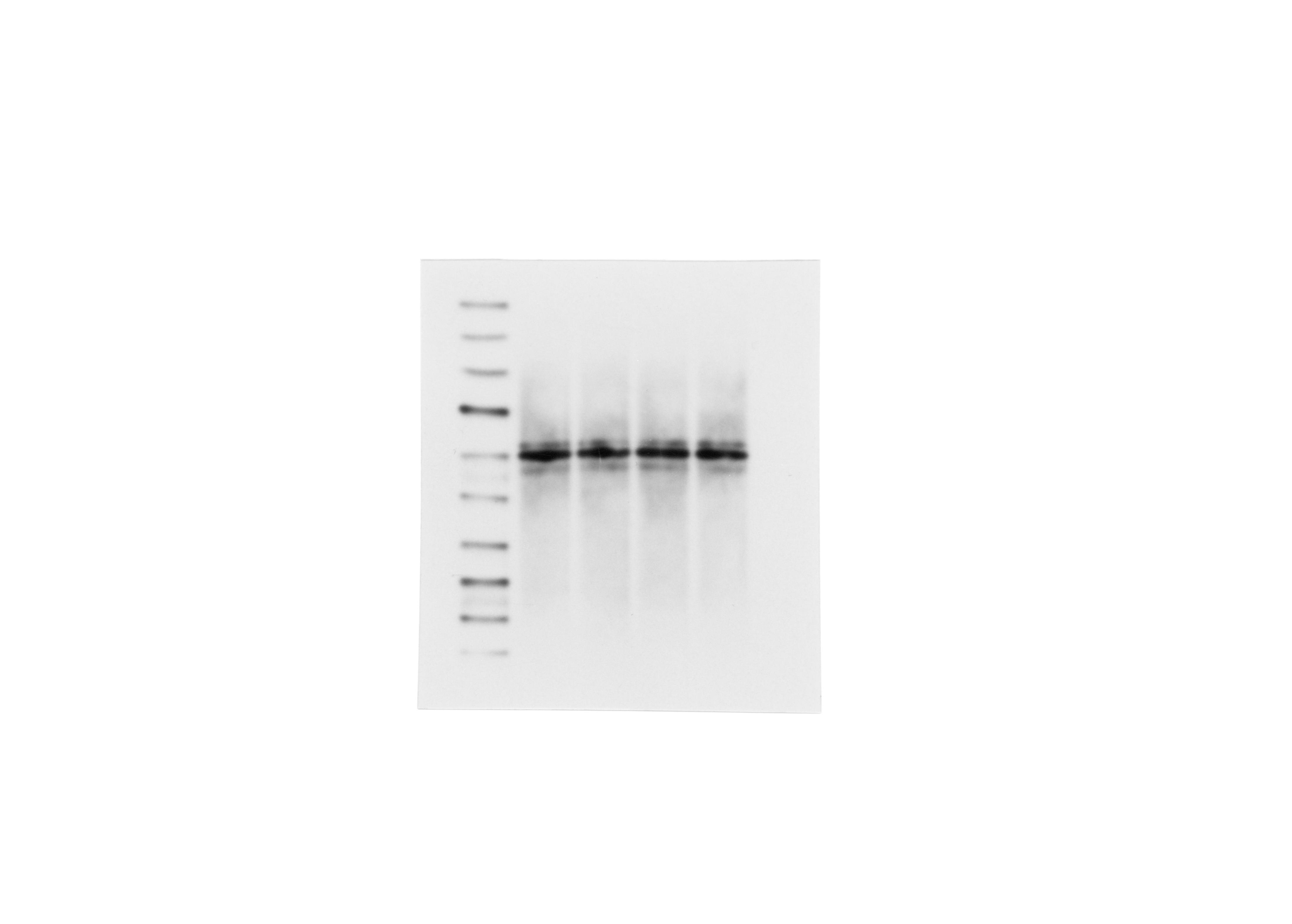

Supplement: Supplementary file 1 — Supplementary Material 1. [file 12672_2026_5064_MOESM1_ESM.zip › Original images for blots and gels/Figure 7 LN229 AKT.tif]

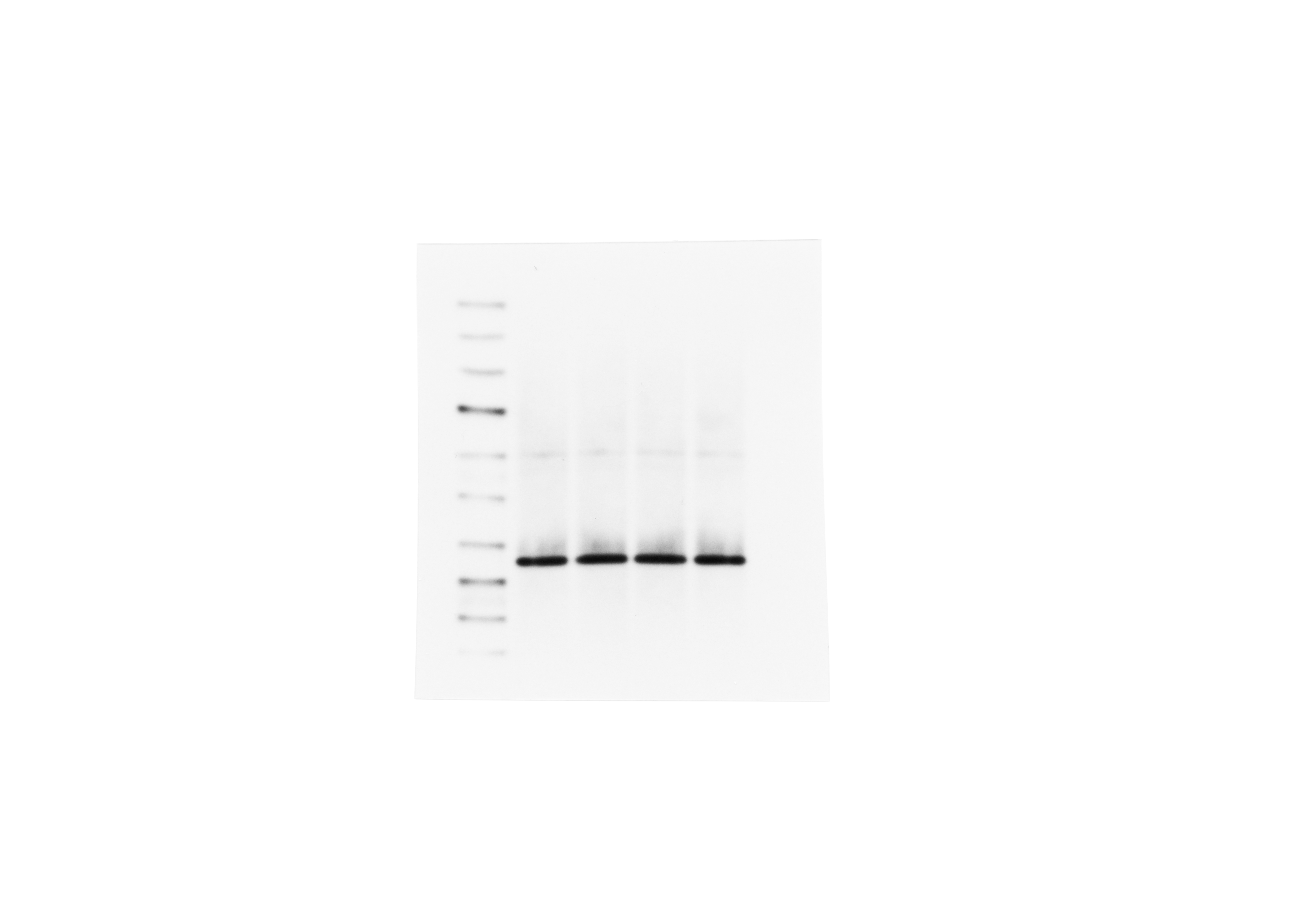

Supplement: Supplementary file 1 — Supplementary Material 1. [file 12672_2026_5064_MOESM1_ESM.zip › Original images for blots and gels/Figure 7 LN229 GAPDH.tif]

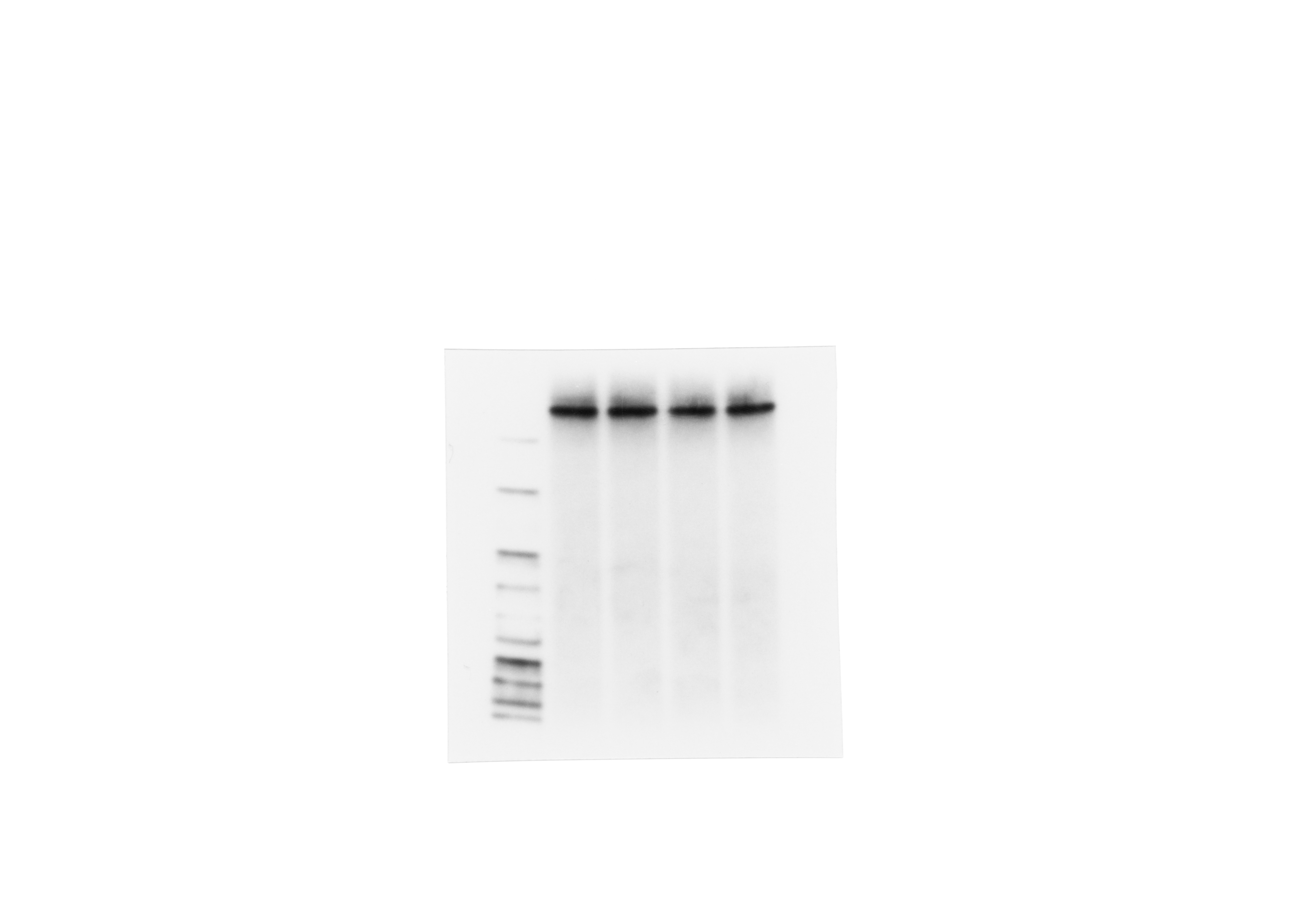

Supplement: Supplementary file 1 — Supplementary Material 1. [file 12672_2026_5064_MOESM1_ESM.zip › Original images for blots and gels/Figure 7 LN229 mTOR.tif]

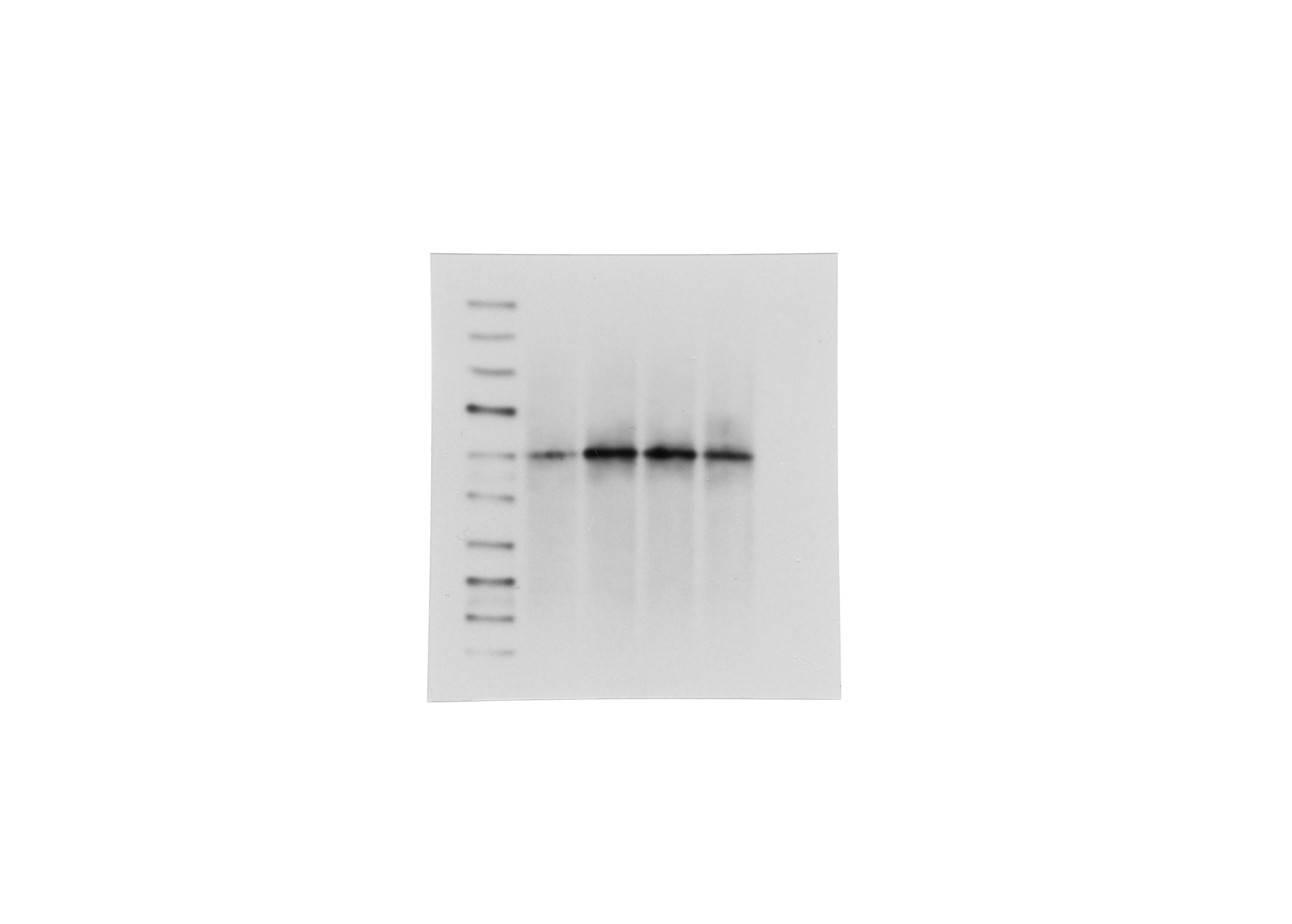

Supplement: Supplementary file 1 — Supplementary Material 1. [file 12672_2026_5064_MOESM1_ESM.zip › Original images for blots and gels/Figure 7 LN229 p-AKT.tif]

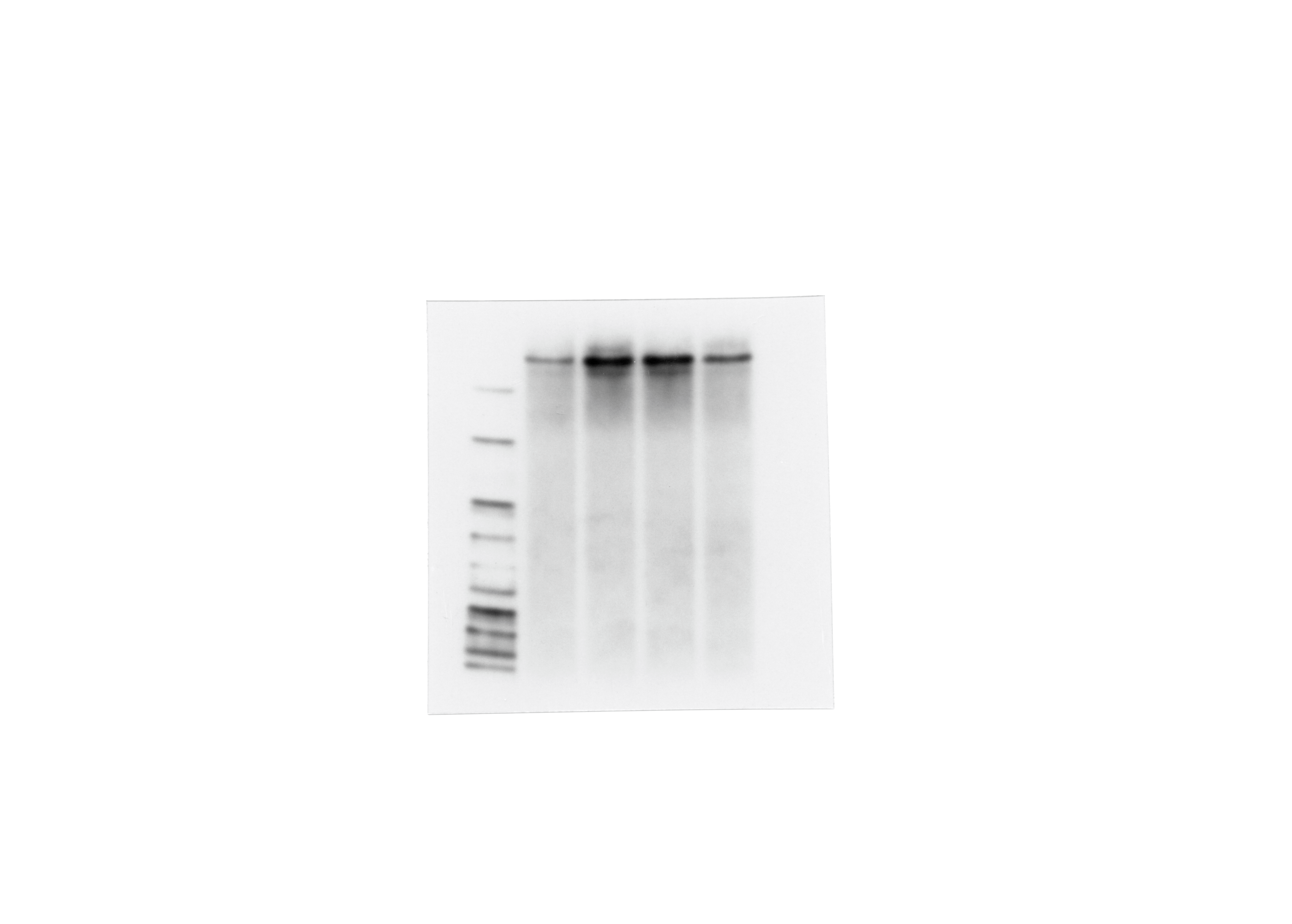

Supplement: Supplementary file 1 — Supplementary Material 1. [file 12672_2026_5064_MOESM1_ESM.zip › Original images for blots and gels/Figure 7 LN229 p-mTOR.tif]

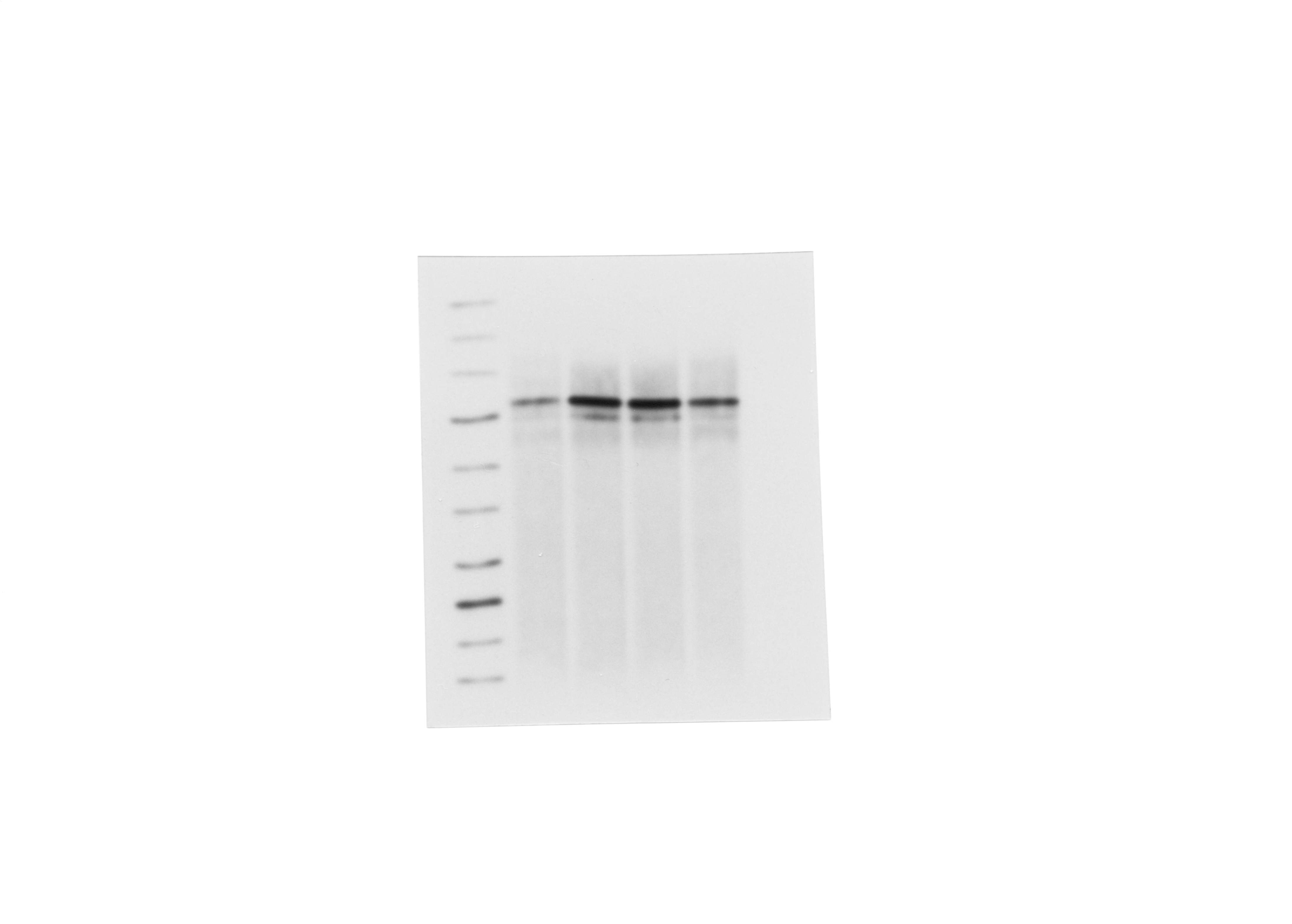

Supplement: Supplementary file 1 — Supplementary Material 1. [file 12672_2026_5064_MOESM1_ESM.zip › Original images for blots and gels/Figure 7 LN229 p-PI3K.tif]

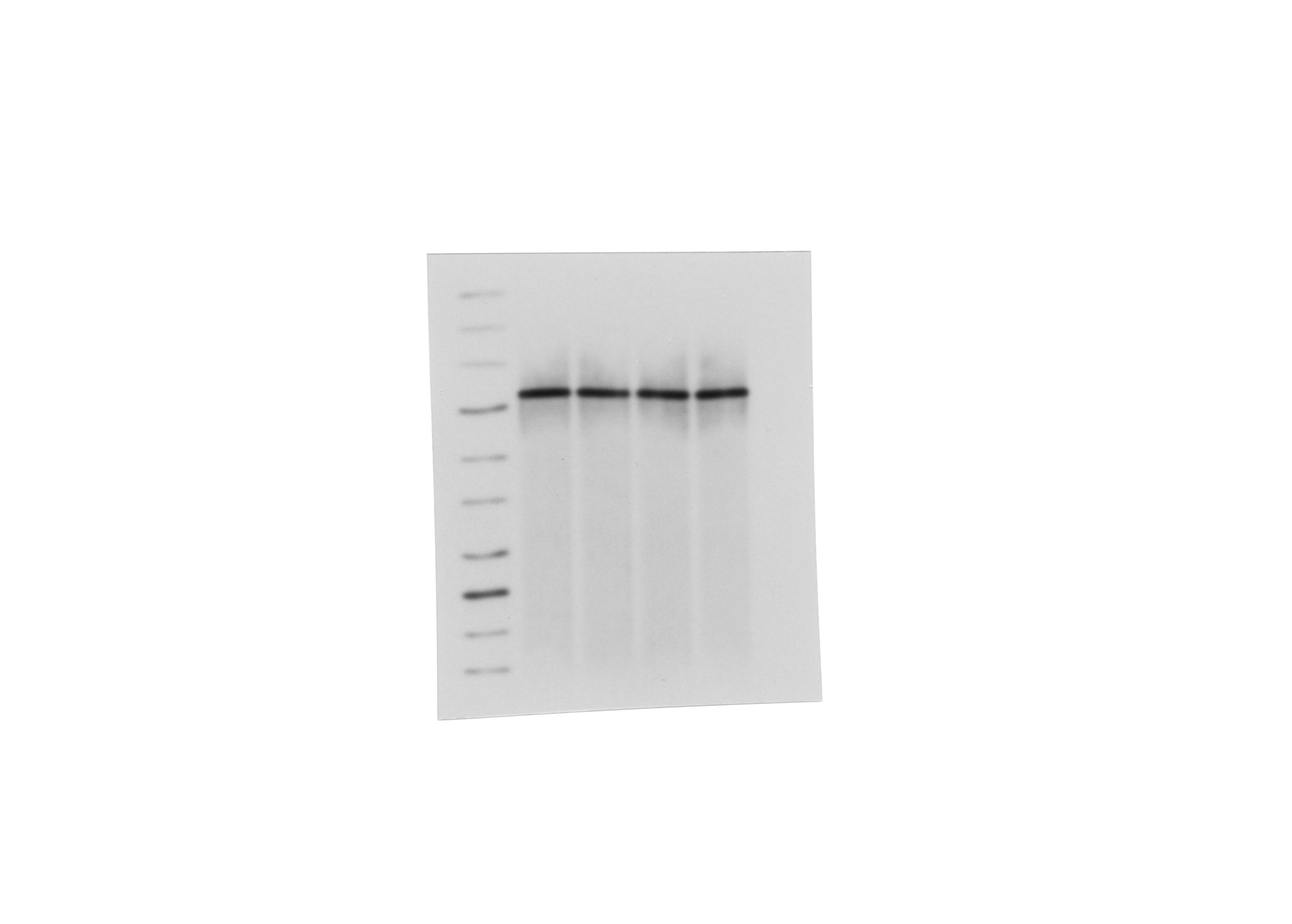

Supplement: Supplementary file 1 — Supplementary Material 1. [file 12672_2026_5064_MOESM1_ESM.zip › Original images for blots and gels/Figure 7 LN229 PI3K.tif]

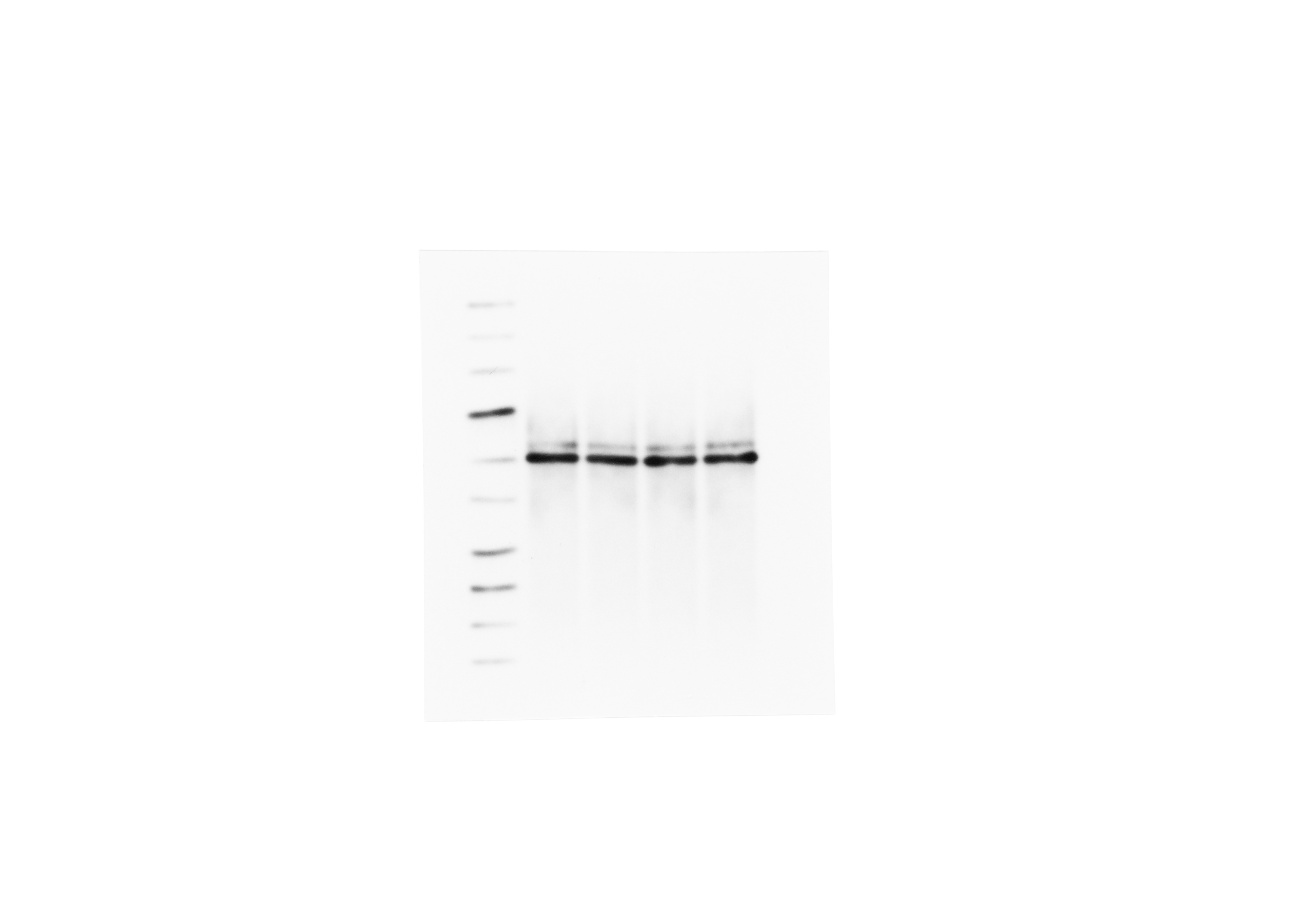

Supplement: Supplementary file 1 — Supplementary Material 1. [file 12672_2026_5064_MOESM1_ESM.zip › Original images for blots and gels/Figure 7 T98G AKT.tif]

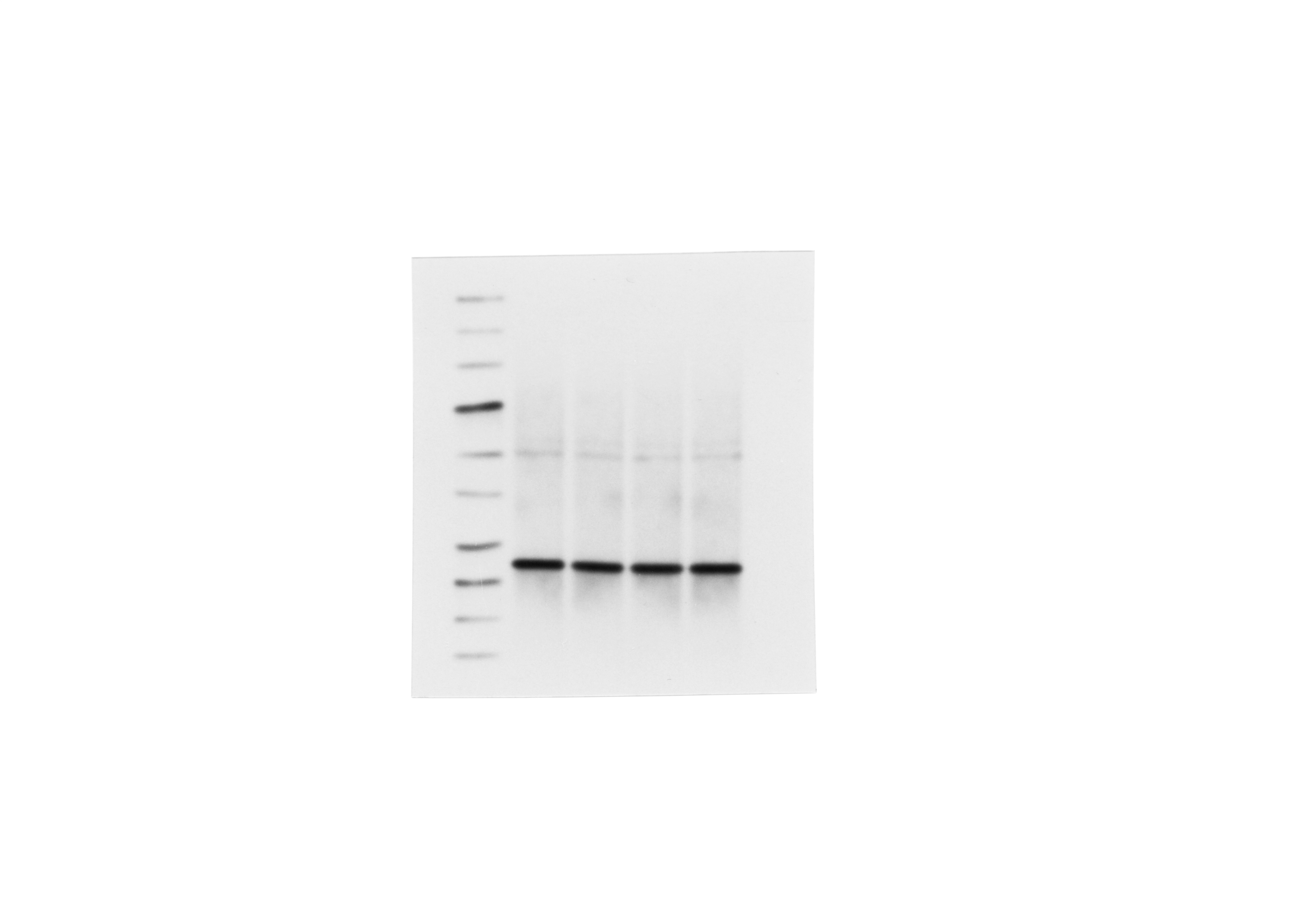

Supplement: Supplementary file 1 — Supplementary Material 1. [file 12672_2026_5064_MOESM1_ESM.zip › Original images for blots and gels/Figure 7 T98G GAPDH.tif]

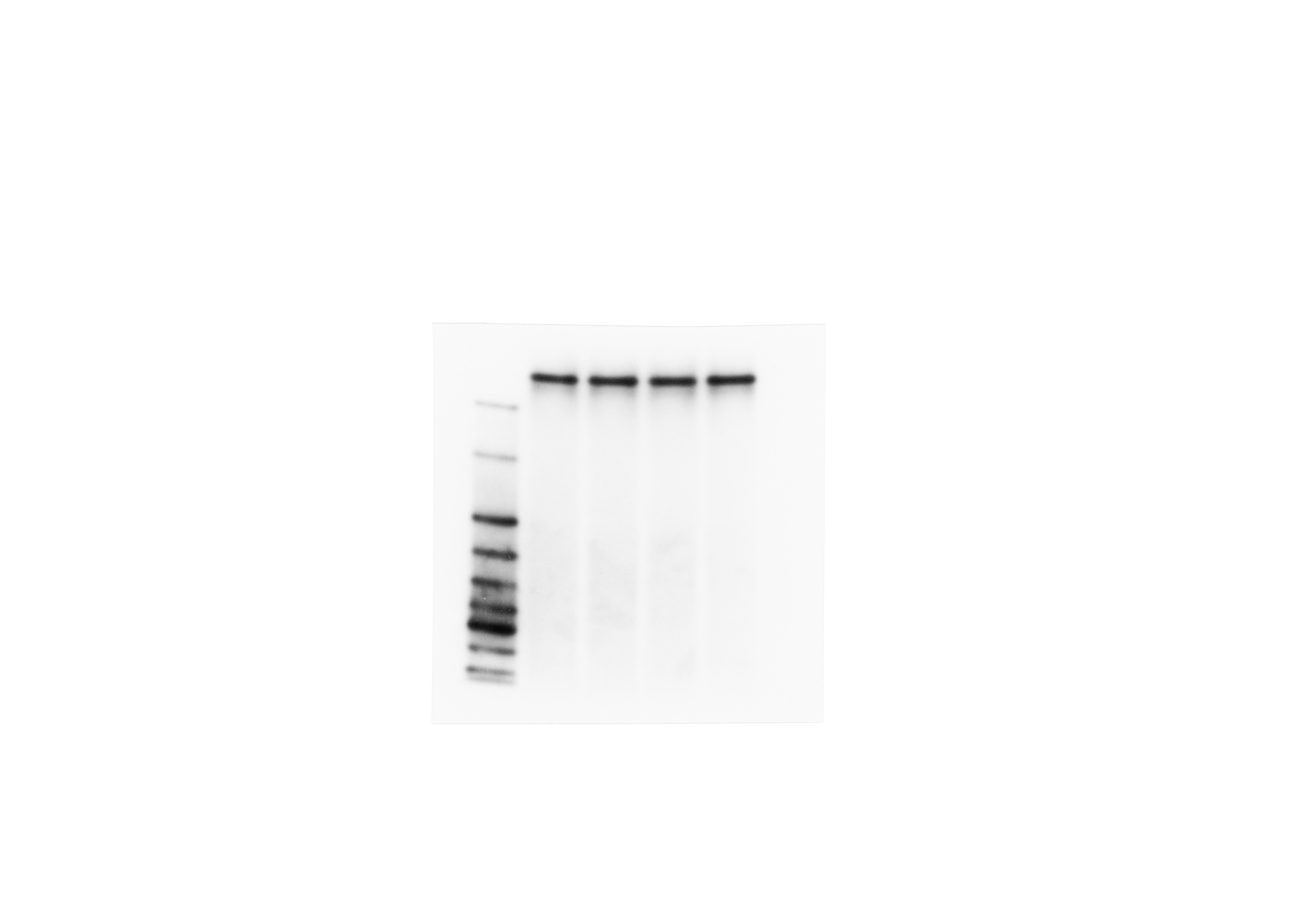

Supplement: Supplementary file 1 — Supplementary Material 1. [file 12672_2026_5064_MOESM1_ESM.zip › Original images for blots and gels/Figure 7 T98G mTOR.tif]

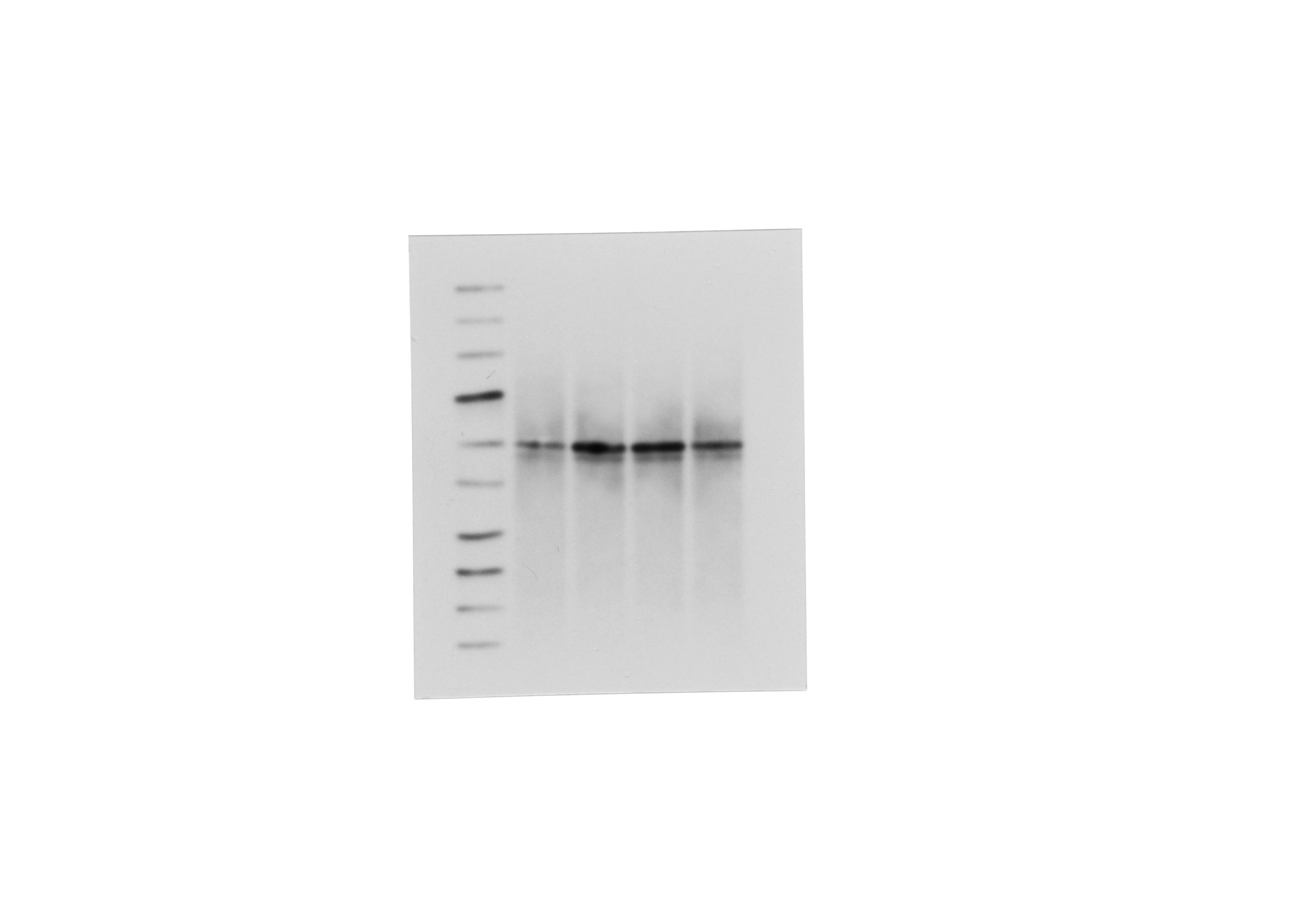

Supplement: Supplementary file 1 — Supplementary Material 1. [file 12672_2026_5064_MOESM1_ESM.zip › Original images for blots and gels/Figure 7 T98G p-AKT.tif]

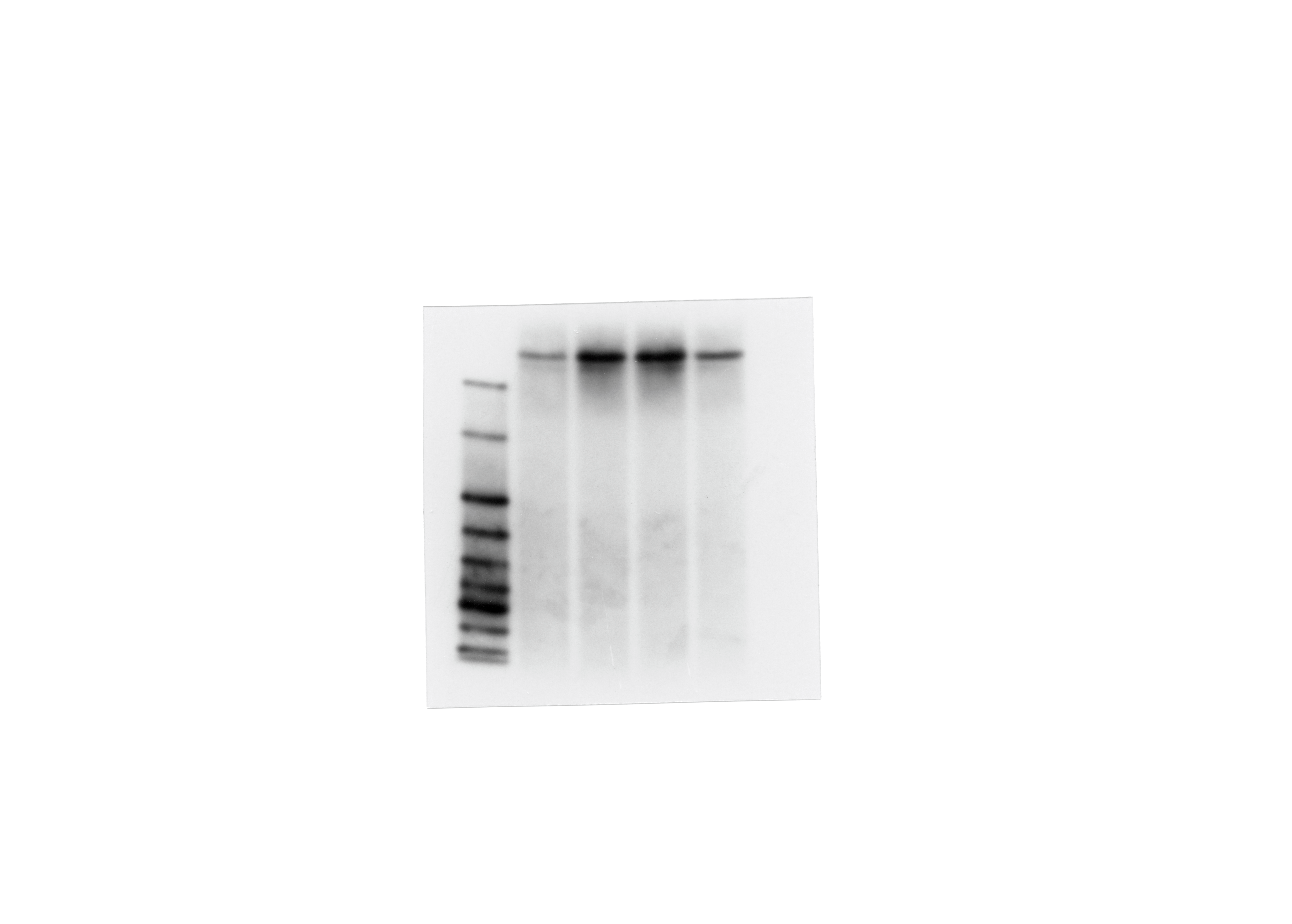

Supplement: Supplementary file 1 — Supplementary Material 1. [file 12672_2026_5064_MOESM1_ESM.zip › Original images for blots and gels/Figure 7 T98G p-mTOR.tif]

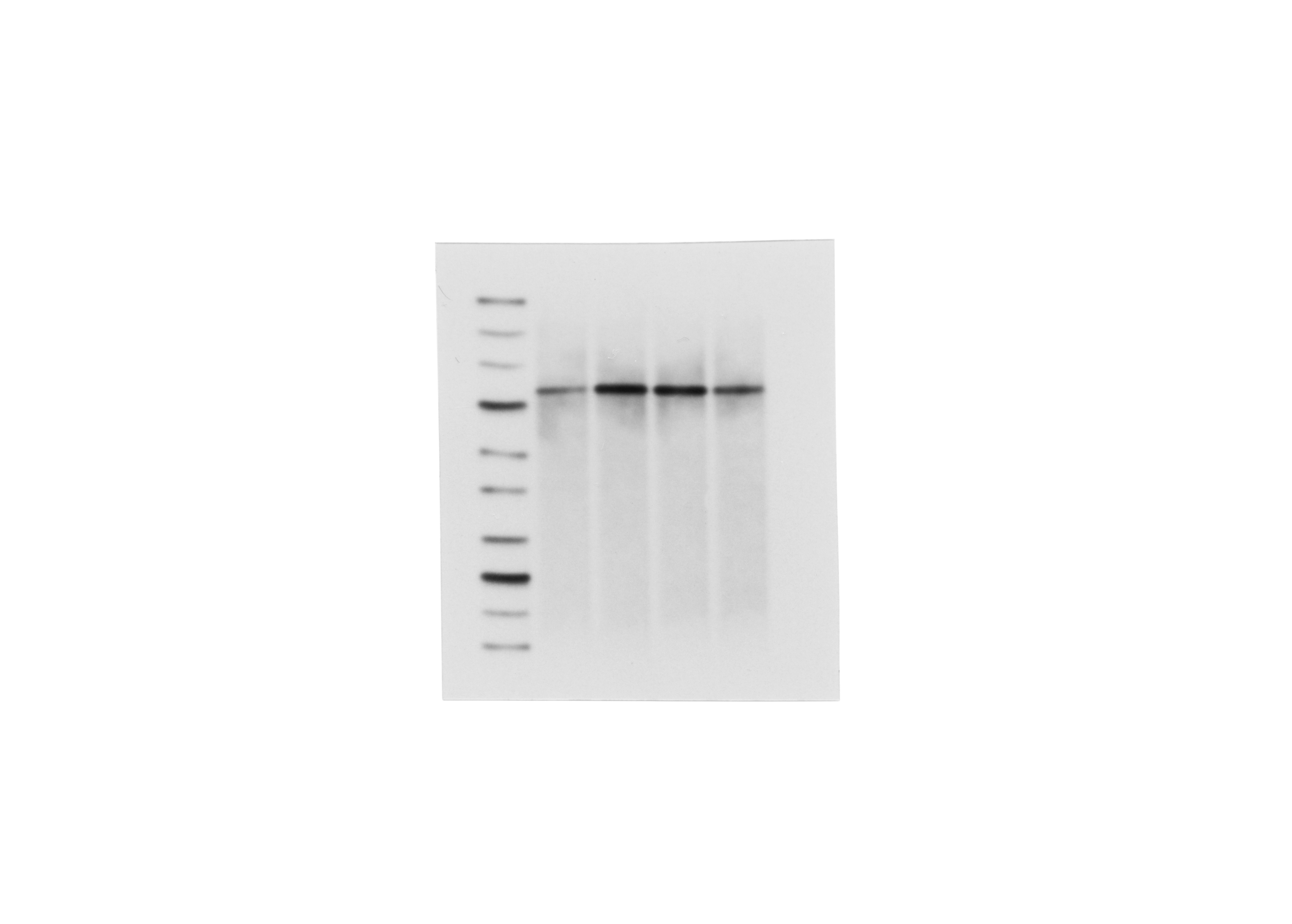

Supplement: Supplementary file 1 — Supplementary Material 1. [file 12672_2026_5064_MOESM1_ESM.zip › Original images for blots and gels/Figure 7 T98G p-PI3K.tif]

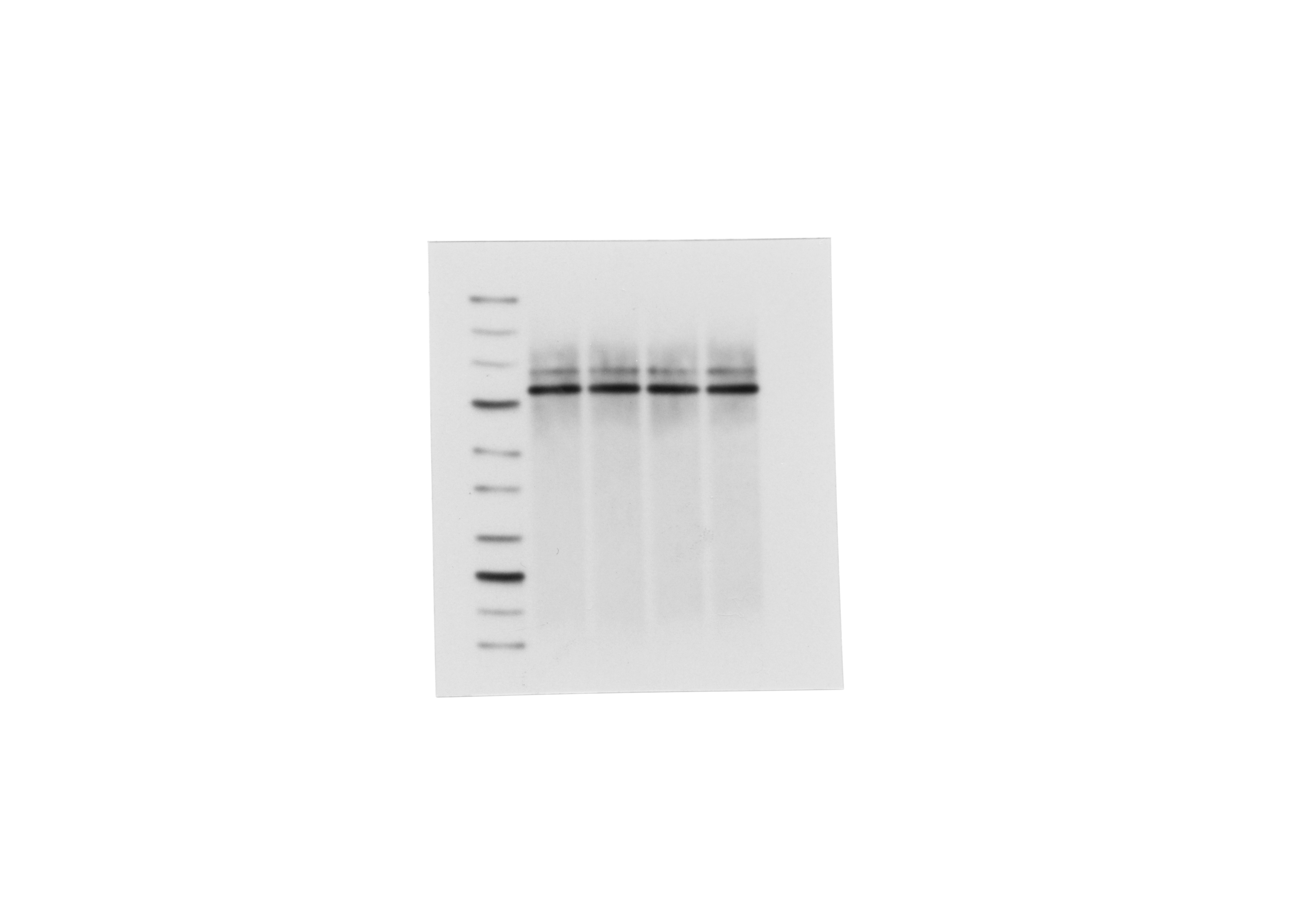

Supplement: Supplementary file 1 — Supplementary Material 1. [file 12672_2026_5064_MOESM1_ESM.zip › Original images for blots and gels/Figure 7 T98G PI3K.tif]
